# A Balanced Multimodal Multi-Task Deep Learning Framework for Robust Patient-Specific Quality Assurance

**DOI:** 10.3390/diagnostics15202555

**Published:** 2025-10-10

**Authors:** Xiaoyang Zeng, Awais Ahmed, Muhammad Hanif Tunio

**Affiliations:** 1 School of Computer Science and Engineering, University of Electronic Science and Technology of China—UESTC, Chengdu 611731, China; 202011081605@std.uestc.edu.cn; 2 School of Computer Science, China West Normal University, Nanchong 637009, China; 3 Institute of Computer Science, Shah Abdul Latif University, Khairpur 66111, Pakistan; hanif.tunio@salu.edu.pk

**Keywords:** trustworthy learning, PSQA, multimodality fusion, modality imbalance, dose difference prediction, Gamma Passing Rate

## Abstract

**Background:** Multimodal Deep learning has emerged as a crucial method for automated patient-specific quality assurance (PSQA) in radiotherapy research. Integrating image-based dose matrices with tabular plan complexity metrics enables more accurate prediction of quality indicators, including the Gamma Passing Rate (GPR) and dose difference (DD). However, modality imbalance remains a significant challenge, as tabular encoders often dominate training, suppressing image encoders and reducing model robustness. This issue becomes more pronounced under task heterogeneity, with GPR prediction relying more on tabular data, whereas dose difference prediction (DDP) depends heavily on image features. **Methods:** We propose **BMMQA** (*Balanced Multi-modal Quality Assurance)*, a novel framework that achieves modality balance by adjusting modality-specific loss factors to control convergence dynamics. The framework introduces four key innovations: (1) task-specific fusion strategies (softmax-weighted attention for GPR regression and spatial cascading for DD prediction); (2) a balancing mechanism supported by Shapley values to quantify modality contributions; (3) a fast network forward mechanism for efficient computation of different modality combinations; and (4) a modality-contribution-based task weighting scheme for multi-task multimodal learning. A large-scale multimodal dataset comprising 1370 IMRT plans was curated in collaboration with Peking Union Medical College Hospital (PUMCH). **Results:** Experimental results demonstrate that, under the standard 2%/3 mm GPR criterion, BMMQA outperforms existing fusion baselines. Under the stricter 2%/2 mm criterion, it achieves a 15.7% reduction in mean absolute error (MAE). The framework also enhances robustness in critical failure cases (GPR < 90%) and achieves a peak SSIM of 0.964 in dose distribution prediction. **Conclusions:** Explicit modality balancing improves predictive accuracy and strengthens clinical trustworthiness by mitigating overreliance on a single modality. This work highlights the importance of addressing modality imbalance for building trustworthy and robust AI systems in PSQA and establishes a pioneering framework for multi-task multimodal learning.

## 1. Introduction

Cancer remains one of the leading causes of mortality worldwide, with radiation therapy serving as a cornerstone in its clinical management. Among advanced techniques, Intensity-Modulated Radiation Therapy (IMRT) has emerged as a pivotal technique in the clinical management of cancer, owing to its advanced capabilities in delivering precise and highly conformal dose distributions that are meticulously tailored to the tumor’s shape and location [1,2]. Nevertheless, the efficacy of IMRT fundamentally depends on the accuracy of dose delivery. This necessitates rigorous verification procedures to ensure the safe and accurate execution of each treatment plan. Patient-specific quality assurance (PSQA) fulfills this need by providing a comprehensive assessment of the treatment plan, thereby ensuring that each treatment plan can be executed both safely and accurately. In clinical practice, two key metrics are widely employed in PSQA: Gamma Passing Rate (GPR) and dose difference (DD) [3,4]. The GPR quantifies the percentage of dose points that satisfy predefined spatial and dosimetric tolerances, providing a coarse-grained measure of treatment plan fidelity. Conversely, the DD offers a detailed spatial distribution of dose discrepancies, yielding fine-grained insight into potential discrepancies between planned and delivered doses. Together, these metrics form the foundation for evaluating and improving the reliability of radiotherapy delivery.

Traditional PSQA methodologies are often labor-intensive, relying significantly on physical measurements and gamma analysis [5,6,7,8,9]. Recent advancements can be classified as follows: manually defined complexity metrics, which tend to have limited clinical relevance; (2) classical machine learning strategies that utilize tabular features, which are constrained by a dependence on low-dimensional representations; and (3) deep learning methods that exploit dose matrices, although these often neglect the advantages of multimodal fusion. Emerging multimodal frameworks seek to enhance the prediction process by integrating dose distribution images with tabular data concerning plan complexity [10,11,12,13,14,15].

Nevertheless, prevailing fusion methodologies often neglect a critical imbalance issue, wherein the tabular modality converges at a significantly swifter pace than its image counterpart due to its lower dimensionality and simpler encoder architecture. This phenomenon is referred to as the *modality imbalance* problem, where the faster-converging modality tends to dominate the joint training process, thereby suppressing the contributions of image-based features and resulting in suboptimal fusion and degraded performance [16]. The issue of modality imbalance in multimodal learning has been extensively explored, especially within classification tasks, where methodologies typically involve the use of an imbalance indicator (such as output logits or gradient magnitudes) alongside an adjustment strategy (including gradient scaling, adaptive sampling, or loss reweighting)  [2,17,18,19,20,21,22,23,24,25,26]. However, PSQA introduces unique challenges as a regression problem characterized by continuous outputs and multi-task requirements (e.g., Gamma Passing Rate prediction and dose difference maps). Unlike classification tasks, regression endeavors exhibit distinct optimization dynamics, rendering traditional balancing strategies less effective.

**Figure 1:**
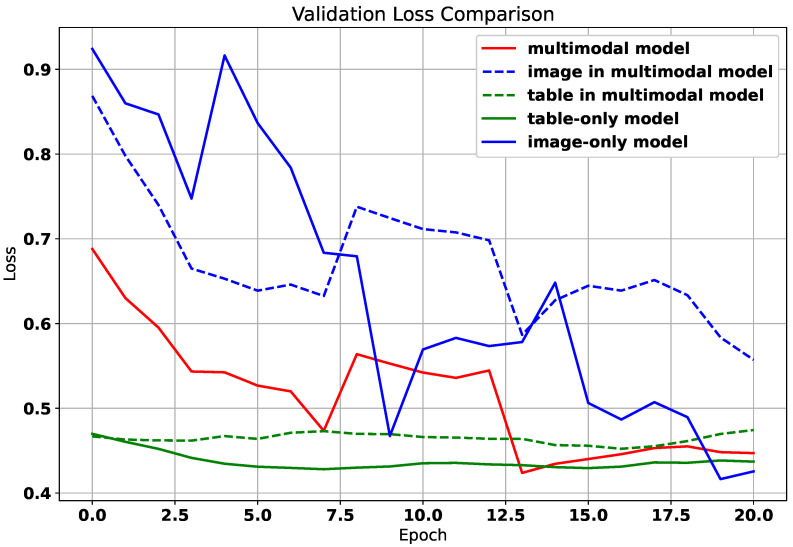
The training loss curve of different modalities.

Figure 1 illustrates an introductory experiment that visualizes the convergence dynamics of different modalities. The solid lines indicate the performance of each modality when trained independently, while the dashed lines represent the performance of unimodal representations during multimodal joint training. Notably, the tabular modality converges as early as the second epoch in both scenarios but subsequently succumbs to overfitting, as evidenced by a gradual increase in loss. In contrast, the image modality continues to improve and remains unconverged even by the twentieth epoch. Importantly, the performance of each modality during joint training consistently lags behind that achieved through independent training. In this experiment, the image modality exhibits the highest performance upon convergence when trained alone, with the blue dashed line significantly surpassing its solid-line counterpart. Additionally, these results reveal a challenge related to modality preference divergence: while the tabular data primarily supports GPR prediction, the image data is more effective for fine-grained dose difference prediction. This heterogeneity complicates joint modeling but also presents an opportunity for targeted balancing.

To mitigate the above-mentioned challenges in multi-task regression for PSQA, we introduce **BMMQA** (Balanced Multi-modal Quality Assurance), a robust framework designed to harmonize modality- and task-specific dynamics. **BMMQA** integrates modality-specific learning rate adjustments to decelerate dominant modality convergence, alongside gradient normalization for stabilized joint training. Central to our approach is a hierarchical fusion mechanism: for coarse-grained GPR regression, we deploy attention-based softmax weighting to adaptively integrate modality embeddings, while spatial concatenation preserves fine-grained structures for dose difference prediction (DDP). Crucially, we derive **Shapley-value-driven imbalance indicators** to quantify modality contributions in continuous-output regression, enabling dynamic task-weight adjustment via gradient normalization and learning rate modulation. This dual-strategy framework not only resolves convergence asynchrony but also optimizes feature collaboration, ensuring clinically reliable predictions. With the help of Peking Union Medical College Hospital, we created a large multimodal dataset of 1370 IMRT plans. Extensive validation on it demonstrates that **BMMQA** achieves state-of-the-art performance, significantly reducing GPR MAE by 15.7% under 2%/2 mm criteria while attaining a peak SSIM of 0.964 for dose distribution prediction.

Our main contributions are as follows:

- **BMMQA** is the first dedicated solution to modality imbalance for radiotherapy QA and multi-task multimodal learning, explicitly addressing convergence asynchrony where tabular features dominate training. A network forward mechanism enabling fast computation of outputs from different modality combinations is also proposed.
- **BMMQA** introduces task-specific fusion protocols: attention-based softmax weighting for GPR regression dynamically balances modality contributions, while spatial concatenation for DDP preserves structural fidelity. This dual-path design resolves representational conflicts between global scalar prediction and fine-grained spatial mapping.
- **BMMQA** develops a theoretically grounded Shapley value framework for quantifying modality contributions, tailored explicitly for continuous-output regression. This framework underpins a dynamic balancing mechanism that modulates training to prevent modality suppression.
- **BMMQA** innovatively addresses the problem of multi-task multimodal learning by introducing a modality-contribution-based task weighting scheme: assigning different task-specific weights according to modality contributions to resolve the modality imbalance problem in multi-task learning.
- Extensive experiments on clinically relevant datasets validate that **BMMQA** outperforms existing methods on both coarse-grained and fine-grained PSQA tasks, significantly improving modality collaboration and task generalization in real clinical settings.

The rest of the study is structured as follows: Section 2 discusses recent related work, followed by Section 3, which describes the dataset and includes relevant discussions, as well as a preliminary task. Further, in Section 4, we present the methodology in a detailed manner, whereas Section 5 provides a thorough discussion of the experimental results. Lastly, Section 6 summarizes this work, and in addition, we highlight the limitations of the current work and discuss future work.

## 2. Related Work

### 2.1. Multimodal PSQA

Multimodal deep learning frameworks have emerged to enhance PSQA by integrating dose distribution matrices (image modality) with plan complexity features (tabular modality), enabling richer and more informative predictions [10,11,12,13,14,15]. These approaches can be broadly categorized based on the stage of modality fusion. Some studies adopt early fusion models, where features from different modalities are extracted separately, concatenated, and then jointly processed by an algorithm, which is typically a machine learning model in existing studies, such as Random Forest [11] or logistic regression [13]. Other works employ a late fusion strategy using ensemble models, where each modality is trained independently to produce its own prediction. These outputs are then combined—commonly via weighted averaging, majority voting, or meta-learning—to yield the final decision. In contrast, other works [10,12] adopt intermediate fusion using end-to-end deep learning architectures, where the multimodal fusion module is jointly optimized with the encoder module during training.

For early fusion methods, existing studies typically employ traditional machine learning algorithms as the modality fusion model. Han et al. [11] incorporated a broad set of tabular features beyond MU values, including MU per control point (MU/CP), the proportion of control points with MU < 3 (%MU/CP < 3), and small segment area per control point (SA/CP). Deep features were extracted from dose matrices using a DenseNet-121 backbone. All modalities—deep, dosimetric, and plan complexity—were independently processed using dimensionality reduction techniques such as PCA and Lasso, and the resulting fused feature vector was used as input to a Random Forest (RF) model for GPR prediction. Li et al. [13] followed a similar approach, performing Lasso-based selection on dosimetric and plan complexity features, and employing logistic regression as the final prediction model. These early fusion approaches generally outperform unimodal baselines and offer relative interpretability. However, their reliance on static feature concatenation and low-dimensional input hinders fine-grained regression tasks such as DDP.

In contrast, ref. [14] proposed a late fusion approach by training two separate support vector machine models—one based on plan complexity features and the other on radiomics features—and combining their outputs to enhance beam-level GPR prediction and classification performance in SBRT (VMAT) plans. The late fusion method is typically included as a baseline in existing studies, such as in the work by Hu et al. [10], where outputs from individual modalities are aggregated at the decision level.

For intermediate fusion methods, Hu et al. [10] proposed a 3D ResNet-based architecture that encodes MLC aperture images to extract spatial features, which are then concatenated with corresponding MU values—a tabular feature representing plan complexity—in a one-to-one manner. This fused representation is used for GPR prediction and is referred to as Feature–Data Fusion (FDF). Huang et al. [12] adopted a similar input structure. Still, they employed a simpler fusion mechanism: compressing the dose matrix into a 99-dimensional vector and concatenating it with a single MU value before passing the result through fully connected layers. Ref. [15] converted delivery log files into MU-weighted fluence maps and used them as inputs to a DenseNet model to predict GPRs under multiple gamma criteria. While these intermediate fusion approaches effectively capture high-dimensional cross-modal embeddings and are well-suited for fine-grained prediction tasks such as dose deviation prediction (DDP), they tend to overlook a key challenge in the PSQA context: the modality imbalance problem, where the tabular modality converges significantly faster than the image modality, leading to sub-optimal optimization and degraded model performance [16]. Table 1 provides a summary of multimodal PSQA methods.

### 2.2. Imbalanced Multimodal Learning

The issue of modality imbalance has also drawn increasing attention in multimodal learning. In intermediate fusion settings, faster-converging modalities often dominate the joint training process, effectively suppressing the contribution of slower, more complex modalities. As a result, rich but harder-to-optimize inputs, such as high-dimensional image features, may be underutilized, leading to sub-optimal multimodal representations. Researchers have proposed modality-specific learning rate scheduling, gradient normalization (e.g., GradNorm), and adaptive weighting of modality contributions during training to mitigate this effect.

The multimodal model should do better than the unimodal model since it works with data that has information from more than one view. However, the widely used multimodal joint training model does not always work well based on existing studies [26], prompting researchers to investigate the reasons for this. Recent studies point out that the jointly trained multimodal model cannot effectively improve the performance with more information as expected due to the discrepancy between modalities [23,26,27,28,29]. Wang et al. [26] found that multiple modalities often converge and generalize at different rates, thus training them jointly with a uniform learning objective is sub-optimal, leading to the multimodal model sometimes is inferior to the unimodal ones. Also, Winterbottom et al. [27] indicated an inherent bias in the TVQA dataset towards the textual subtitle modality. Besides the empirical observation, Huang et al. [29] further theoretically proved that the jointly trained multimodal model cannot efficiently learn features of all modalities, and only a subset of them can capture sufficient representation. They called this process “Modality Competition”. Recently, several methods have emerged attempting to alleviate this problem [23,24,26,30]. Wang et al. [26] proposed to add additional unimodal loss functions besides the original multimodal objective to balance the training of each modality. Du et al. [30] utilized well-trained unimodal encoders to improve the multimodal model by knowledge distillation. Wu et al. [23] found out how quickly the model learns from one type of input compared to the other types of input, and then proposed to guide the model to learn from previously underutilized modalities. Wei et al. [31] introduced a Shapley-based sample-level modality valuation metric to observe and alleviate the fine-grained modality discrepancy. Wei et al. [32] further considered the possible limited capacity of modality and utilized the re-initialization strategy to control unimodal learning. In contrast, Yang et al. [33] focused on the influence of imbalanced multimodal learning on multimodal robustness, and proposed a robustness enhancement strategy.

While these methods have improved multimodal learning, a comprehensive analysis of imbalanced multimodal learning is still lacking. Most previous studies have addressed modality imbalance in single-task scenarios. However, in real-world applications—such as PSQA in the medical domain—multimodal models are often designed for multi-task settings, where modality imbalance typically results in the degradation of the performance of an entire task. This is the central problem our work aims to address.

## 3. Foundations

This section establishes the methodological foundations for our study.

### 3.1. Clinically Validated Data Curation

For this study, we utilized the private data set obtained in collaboration with the Department of “Radiation Oncology” at “Peking Union Medical College Hospital” https://www.pumch.cn/en/introduction.html, accessed on 3 October 2025. The dataset was collected between December 2020 and July 2021. It comprises 210 FF-IMRT treatment plans, encompassing 1370 beam fields for various treatment sites, and the treatment plans by site are detailed in Table 2.

All plans were generated using Eclipse TPS version 15.6 (Varian Medical System) and delivered via Halcyon 2.0 linac with SX2 dual-layer MLC. The sliding window was employed for plan optimization, with dose calculation performed using the Anisotropic Analytic Algorithm (AAA, version 15.6.06) at a 2.5 mm grid resolution.

Under the TG-218 report recommendation [34], PSQA measurements were conducted before treatment delivery using actual beam angles and employing portal dosimetry. Dose calibration was performed daily before data collection. Gamma analyses were executed with criteria of 1%/1 mm, 2%/2 mm, and 2%/3 mm at a 10% threshold of maximum dose, analyzing only points with doses greater than 10% of the global maximum dose per beam, as detailed in Table 3. The gamma analyses were carried out in absolute dose mode, applying global normalization to the results. The treatment planning system calculated fluence maps, exported in DICOM format, which served as inputs for the deep learning network. The raw fluence maps underwent preprocessing to standardize input data: resampling to 1 × 1 ${\mathrm{mm}}^{2}$ resolution, cropping to 512 × 512 pixels, and Min-Max normalization to scale pixel values to [0, 1]. This comprehensive dataset, alongside rigorous PSQA measurements, underpins the proposed Virtual Dose Verification.

**Figure 2:**
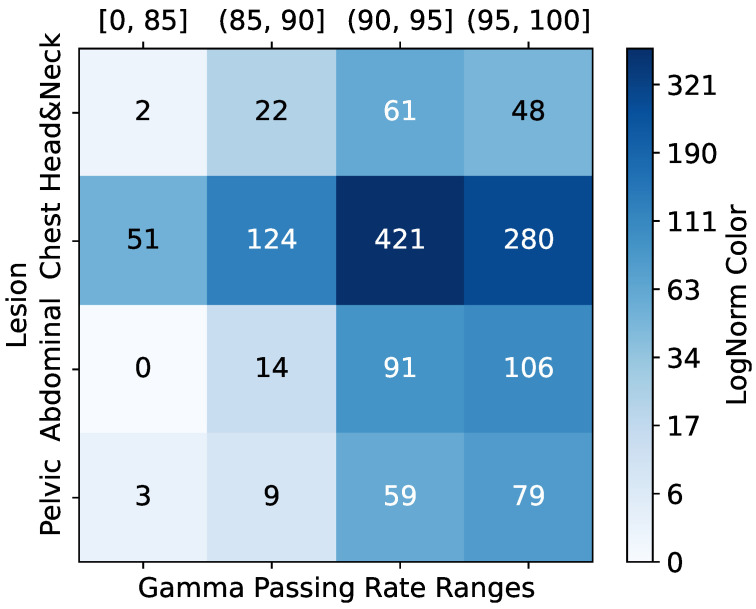
Sample GPR and lesion distribution.

**Figure 3:**
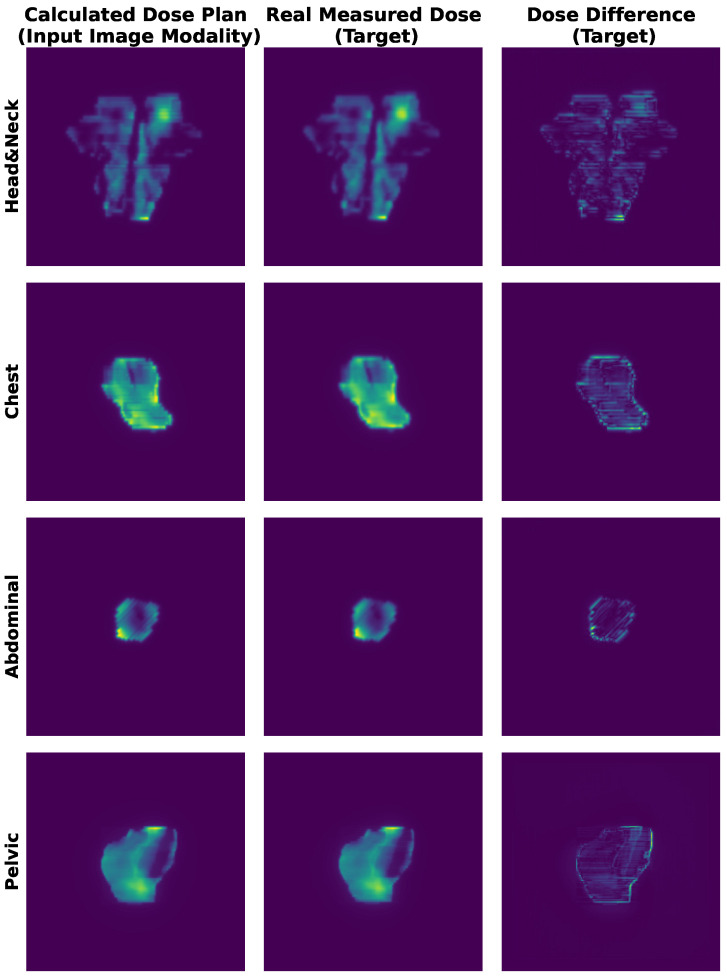
Representative IMRT QA results for four treatment plans. (**Left**) TPS-calculated dose distribution. (**Middle**) Measured dose via portal dosimetry. (**Right**) Absolute dose differences. Pixel intensity corresponds to delivered dose (Gy).

The dataset’s GPR distribution, categorized into clinical intervals (<85%, 85–90%, 90–95%, >95%), revealed site-specific trends as shown in Figure 2; for example, head and neck (H&N) plans exhibited higher complexity (51 beams below 85% GPR). In comparison, chest (C) plans demonstrated optimal compliance (106 beams > 95% GPR). Qualitative PSQA validation as shown in Figure 3, further compared TPS-calculated doses, measured doses, and absolute dose differences across four representative IMRT plans, with the pixel intensity (darker = higher dose Gy) highlighting discrepancies, particularly in high-gradient regions. These visualizations collectively underscore the dataset’s variability and the critical role of rigorous QA, especially for an anatomically complex site like H&N.

### 3.2. Task Formulation

In our PSQA task, each input sample *X* comprises two modalities: an image modality $X^{\mathrm{img}}$ and a tabular modality $X^{\mathrm{tab}}$. The image modality $X^{\mathrm{img}}$ is a 512×512 (H×W) matrix representing relative dose intensity values normalized to the range [0,1]. The tabular modality $X^{\mathrm{tab}}$ is a 33-dimensional vector composed of plan complexity metrics.

We consider two prediction tasks. The first is GPR prediction, where the target variable $Y_{\mathrm{gpr}}$ is a three-dimensional vector corresponding to three standard clinical criteria. The second is DDP, where the target variable $Y_{\mathrm{ddp}}$ is a 512×512 matrix aligned with the spatial resolution of the image input.

Table 4 summarizes the mathematical symbols used throughout this study. Superscripts indicate modalities (e.g., img, tab), while subscripts refer to task-specific roles (e.g., gpr, ddp). These definitions provide a consistent multimodal framework for modeling the PSQA task, enabling both coarse-grained and fine-grained prediction objectives.

### 3.3. Shapley Value

The *Shapley value* [42] was originally formulated in coalitional game theory to fairly allocate the total payoff among players based on their contributions. A multi-player game is typically described by a real-valued utility function *V* defined over a set of players N:={1,…,n}. Players can form various coalitions (i.e., subsets of N), and their collective interactions can be either cooperative or competitive.

To quantify the contribution of a specific player *i*, consider a coalition S⊆N∖{i}. The marginal contribution of player *i* to coalition *S* is defined as

$$m_{i}\left(S;V\right)=V\left(S\cup \left\{i\right\}\right)-V\left(S\right).$$

Since a player’s influence depends on all possible coalitions it may join, the Shapley value $\phi_{i}$ is defined as the average of its marginal contributions across all such subsets, weighted by the size of the coalitions:

$$\phi_{i}\left(N;V\right):=\sum_{S\subseteq N\setminus \{i\}}\frac{|S|!(n-|S|-1)!}{n!}\;m_{i}\left(S;V\right).$$

A fair allocation of payouts in cooperative game theory is characterized by four fundamental axioms: *Efficiency*, *Symmetry*, *Dummy*, and *Additivity*. Among attribution methods, the Shapley value is uniquely defined as the only solution that satisfies all four properties [43]. More formally:

**Efficiency:** The total payout is fully distributed among all players, i.e., $\sum_{i}\phi_{i}=V\left(N\right)-V\left(⌀\right)$, where V(⌀) represents the utility of the empty set (baseline with no players).

**Symmetry:** If two players *i* and *j* contribute equally to every coalition, that is, V(S∪{i})=V(S∪{j}) for all S⊆N∖{i,j}, then their attributions must be identical: $\phi_{i}=\phi_{j}$.

**Dummy:** A player *i* is considered a dummy if its inclusion does not affect any coalition beyond its standalone contribution, i.e., V(S∪{i})=V(S)+V({i}) for all S⊆N∖{i}. In this case, the player’s attribution equals its value: $\phi_{i}=V\left(\left\{i\right\}\right)$.

**Additivity:** For any two games with utility functions *V* and *W*, their combined game is defined as U(S)=V(S)+W(S) for all S⊆N. The attribution in the combined game satisfies linearity: $\phi_{i}\left(U\right)=\phi_{i}\left(V\right)+\phi_{i}\left(W\right)$.

These axioms collectively define the criteria for fair attribution, and it has been mathematically proven that the Shapley value is the only allocation method that satisfies them all [44].

Game-theoretic principles have been widely adopted in machine learning research [45,46,47]. For instance, game theory has been used to explain the effectiveness of ensemble methods such as AdaBoost [45]. In a related line of work, Hu et al. [48] utilize the Shapley value to assess the overall contribution of individual modalities across an entire dataset. However, their approach does not address modality contributions at the sample level and lacks a deeper analysis of underperforming modalities.

In this work, we propose a method for *sample-level modality valuation*, providing a more fine-grained understanding of modality importance. Furthermore, we analyze and propose solutions to mitigate the impact of low-contributing modalities.

## 4. Methodology

### 4.1. Overview

**Figure 4:**
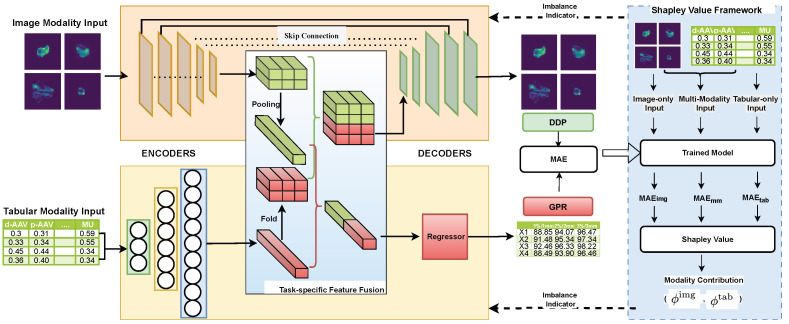
Architecture diagram of the proposed **BMMQA**.

As Figure 4 depicts, the **BMMQA** framework integrates four modular components for robust radiotherapy quality assurance: Input Layer processes dual-modality data (spatial dose matrices and tabular complexity metrics); modality encoders transform inputs into high-level features via pre-trained image encoding and MLP-based tabular embedding; task-specific feature fusion dynamically balances contributions using attention-based weighting for GPR regression and spatial concatenation for DDP mapping; and decoders generate task-specific predictions (scalar GPR values and 512 × 512 DDP matrices via U-Net decoding). This structured pipeline ensures modality-aware feature collaboration while preserving spatial integrity for clinical reliability.

To further mitigate modality imbalance during training, **BMMQA** incorporates a dynamic balancing mechanism that integrates multiple synergistic components: (1) Task-specific fusion strategies: a softmax-weighted attention mechanism is used for GPR regression, while spatial cascading is applied for DD prediction. (2) A theoretically supported balancing mechanism: Shapley values, particularly suited for multi-task regression, are employed to quantify the contribution of each modality. (3) A fast network forward mechanism designed to compute the output of different modality combinations efficiently. (4) A modality-contribution-based task weighting scheme developed explicitly for multi-task multimodal learning: assigning different task-specific weights according to modality contributions to address the modality imbalance problem in multi-task learning.

This closed-loop design enhances robustness, particularly in critical failure cases (e.g., GPR < 90%), by preserving feature collaboration integrity across both coarse-grained and fine-grained PSQA tasks.

### 4.2. Modality Encoders

The model input consists of two modalities: **image modality** dose plan arrays $X^{img}$, and **tabular modality** plan complexity metrics $X^{tab}$. The image modality is passed through a convolutional or Transformer-based pretrained encoder (e.g., ResNet) to extract spatial features, yielding a high-dimensional embedding $Z^{\mathrm{img}}\in R^{N_{c}\times W\times H}$, where $N_{c}$ is the number of channels, and W,H denote the spatial dimensions.

To ensure dimensional compatibility for subsequent fusion, the tabular modality is processed by an MLP encoder to extract structured feature embeddings. These embeddings are initially represented as $Z^{\mathrm{tab}}\in R^{(N_{t}\cdot W\cdot H)\times 1}$, where · denotes scalar multiplication.

The encoding process can be formalized as:

(1) $$\begin{array}{cc} Z^{\mathrm{img}} & ={\mathrm{Enc}}^{\mathrm{img}}\left(X^{\mathrm{img}}\right), \end{array}$$

(2) $$\begin{array}{cc} Z^{\mathrm{tab}} & ={\mathrm{Enc}}^{\mathrm{tab}}\left(X^{\mathrm{tab}}\right), \end{array}$$

### 4.3. Task-Specific Modality Fusion

The intrinsic task heterogeneity between Gamma Passing Rate (GPR) regression (scalar output) and dose difference prediction (DDP) (spatial matrix output) necessitates divergent fusion strategies. This duality manifests in two critical dimensions: **Representational Conflict**: the GPR requires compact, modality-agnostic feature abstraction for global dose compliance assessment, while DD demands high-resolution spatial preservation for localized discrepancy mapping. **Optimization Incompatibility**: Naive uniform fusion (e.g., concatenation or averaging) causes representational interference—spatial details critical for DD are diluted by low-dimensional tabular features optimized for GPRs. To resolve this duality, **BMMQA** introduces a task-specific modality fusion mechanism, **GPR-Oriented Fusion** prioritizes attention-based modality weighting to distill task-relevant signals while suppressing noise through differentiable softmax gates. **DDP-Oriented Fusion** maintains structural integrity via channel-wise spatial alignment, preserving high-resolution image embeddings without destructive compression.

#### 4.3.1. GPR-Oriented Fusion

In the GPR task, the objective is to predict coarse-grained scalar values rather than spatial maps. We first apply spatial pooling to the high-dimensional feature maps extracted from the image modality to align the modalities for this point-wise regression. This yields a compact image embedding that is dimensionally compatible with the tabular embedding. The two embeddings are then concatenated to form a joint representation for regression.

Due to the opaque nature of deep networks, assessing **modality imbalance** during training requires visibility into how each modality contributes to the final output. While prior work has proposed feed-forward architectures that isolate modal contributions, these are typically designed for classification tasks, where the output is normalized via softmax. In regression tasks like PSQA, the outputs are not inherently normalized, which leads to *scale shift* problems when isolating modality contributions, rendering many imbalance indicators ineffective.

To address this, we adopt an attention-based fusion mechanism on the modality embeddings. Instead of normalizing at the output layer, we apply a softmax weighting to the modality embeddings during fusion. This design enables transparent and differentiable attribution of modality contributions, making it suitable for imbalance assessment in regression settings.

Each modality embedding is projected to a scalar score *s* via a learnable linear transformation:

(3) $$s^{\mathrm{img}}=W_{s}^{\mathrm{img}⊤}Z^{\mathrm{img}},\;s^{\mathrm{tab}}=W_{s}^{\mathrm{tab}⊤}Z^{\mathrm{tab}}$$

For notational simplicity, we write $W^{⊤}Z^{\mathrm{img}}$ as a matrix multiplication, where $Z^{\mathrm{img}}\in R^{C\times H\times W}$ are feature maps. In practice, spatial pooling (e.g., global average pooling) is applied to ensure shape compatibility before the projection. This convention is adopted throughout the remainder of this paper.

The scores *s* are normalized using a softmax function to obtain attention weights α that reflect the relative contribution of each modality:

(4) $$\begin{array}{cc} \alpha^{\mathrm{img}} & =\frac{\exp(s^{\mathrm{img}})}{\exp\left(s^{\mathrm{img}}\right)+\exp\left(s^{\mathrm{tab}}\right)}\\ \alpha^{\mathrm{tab}} & =\frac{\exp(s^{\mathrm{tab}})}{\exp\left(s^{\mathrm{img}}\right)+\exp\left(s^{\mathrm{tab}}\right)} \end{array}$$

The final fused representation is computed as a weighted combination of the modality-specific embeddings:

(5) $$Z_{\mathrm{gpr}}=Z_{\mathrm{gpr}}^{\left(\mathrm{img},\mathrm{tab}\right)}=\alpha^{\mathrm{img}}\cdot \left(W^{\mathrm{img}⊤}Z^{\mathrm{img}}\right)+\alpha^{\mathrm{tab}}\cdot \left(W^{\mathrm{tab}⊤}Z^{\mathrm{tab}}\right)$$

We denote $Z_{\mathrm{gpr}}^{\left(\mathrm{img},\mathrm{tab}\right)}$ as the joint representation derived from both image and tabular modalities. For clarity, we simplify this as $Z_{\mathrm{gpr}}$ when both modalities are present.

#### 4.3.2. DDP-Oriented Fusion

Since dose difference prediction requires generating spatial outputs, it is essential to preserve spatial information from the high-dimensional image modality. We argue that applying modality attention in this task is suboptimal. Therefore, this stage does not use the attention-based modality fusion mechanism described in the previous section.

To align the tabular features with the spatial structure of the image features, we first fold the tabular embedding vector $Z^{\mathrm{tab}}\in R^{N_{t}\cdot H\cdot W}$ into feature maps $R^{N_{t}\times H\times W}$. The reshaped $Z^{\mathrm{tab}}$ is then concatenated with the image feature map $Z^{\mathrm{img}}$ along the channel dimension to form the joint representation $Z_{\mathrm{ddp}}\in R^{(N_{c}+N_{t})\times H\times W}$. Notably, the number of tabular channels $N_{t}$ is intentionally set much smaller than the number of image channels $N_{c}$, reflecting our prior assumption that the DDP task should place greater emphasis on the image modality, which inherently preserves spatial information.

(6) $$Z_{\mathrm{ddp}}=\mathrm{Concat}\left(\mathrm{Fold}\left(Z^{\mathrm{tab}}\right),Z^{\mathrm{img}}\right)$$

### 4.4. Task-Specific Decoders

The fused multimodal representations ($Z_{gpr}$ and $Z_{ddp}$) require distinct decoding pathways tailored to the dimensional characteristics of their respective prediction tasks. The scalar output of GPRs necessitates progressive feature compression through fully connected layers, which transform high-dimensional inputs into compact regression targets. Conversely, the matrix output of DDP requires the maintenance of topological consistency through convolutional decoders that preserve spatial relationships.

#### 4.4.1. GPR Decoder

After attention-based fusion, the resulting joint representation is further processed to enhance non-linearity and enable prediction. Specifically, the fused vector $Z_{\mathrm{gpr}}$ is first passed through a ReLU activation function, followed by an MLP to generate the final GPR prediction:

(7) $${\hat{Y}}_{\mathrm{gpr}}={\hat{Y}}_{\mathrm{gpr}}^{\left(img,tab\right)}=f_{gpr}^{\left(img,tab\right)}\left(Z_{gpr}\right)=\mathrm{MLP}\left(\mathrm{ReLU}\left(Z_{\mathrm{gpr}}\right)\right)$$

Here, the MLP consists of one or more fully connected layers that transform the activated fusion output into a scalar regression value. This structure allows the model to capture complex interactions between modalities beyond the initial linear fusion.

To optimize the GPR regression model, we adopt the mean absolute error (MAE) as the training loss:

(8) $$L_{gpr}=\mathrm{MAE}\left(Y_{gpr},{\hat{Y}}_{gpr}\right)=\frac{1}{n}\sum_{i=1}^{n}\left|{\left(Y_{gpr}\right)}_{i}-{\left({\hat{Y}}_{gpr}\right)}_{i}\right|$$

#### 4.4.2. Dose Difference Decoder

To generate matrix-form outputs, we adopt a U-Net-style decoder, commonly used in image segmentation tasks. The joint representation $Z_{\mathrm{ddp}}$ is passed through a series of convolutional layers that progressively reduce the channel dimension to 1, resulting in a continuous-valued prediction of the dose difference map ${\hat{Y}}_{\mathrm{ddp}}$.

(9) $${\hat{Y}}_{\mathrm{ddp}}=f_{\mathrm{ddp}}\left(Z_{\mathrm{ddp}}\right)$$

To ensure a consistent loss scale for multi-task training, we also adopt the MAE as the training loss for the DDP matrix regression task:

(10) $$L_{\mathrm{ddp}}=\mathrm{MAE}\left(Y_{\mathrm{ddp}},{\hat{Y}}_{\mathrm{ddp}}\right)=\frac{1}{n}\sum_{i=1}^{n}\left|{\left(Y_{\mathrm{ddp}}\right)}_{i}-{\left({\hat{Y}}_{\mathrm{ddp}}\right)}_{i}\right|$$

Here, $Y_{\mathrm{ddp}}\in R^{H\times W}$ and ${\hat{Y}}_{\mathrm{ddp}}\in R^{H\times W}$ are the ground-truth and predicted dose difference matrices, respectively, and n=H×W denotes the total number of spatial elements.

### 4.5. Dynamic Balancing Mechanism

The persistent dominance of tabular modalities during joint optimization necessitates a rigorous quantification framework to address two fundamental manifestations of imbalance: (1) gradient suppression, where earlier-converging modalities produce vanishing gradients that hinder updates to slower modality encoders, and (2) representational collapse, wherein non-dominant modalities fail to achieve optimal capacity due to inhibited feature learning. To mitigate these issues, we base our balancing mechanism on coalitional game theory, utilizing Shapley values to provide an axiomatically fair attribution metric suitable for continuous-output regression scenarios.

#### 4.5.1. Shapley-Based Imbalance Indicator

To reveal the extent of modality imbalance, we formalize modality ablation within the feed-forward process by defining the following single-modality and null-modality variants:

**Image-only Forward.** When only the image modality is used, the fusion reduces to:

(11) $$Z_{\mathrm{gpr}}^{\mathrm{img}}=1\cdot \left(W^{\mathrm{img}⊤}Z^{\mathrm{img}}\right)+0\cdot \left(W^{\mathrm{tab}⊤}Z^{\mathrm{tab}}\right)=W^{\mathrm{img}⊤}Z^{\mathrm{img}}$$

**Tabular-only Forward** Similarly, when only the tabular modality is used, the fused representation becomes:

(12) $$Z_{\mathrm{gpr}}^{\mathrm{tab}}=0\cdot \left(W^{\mathrm{img}⊤}Z^{\mathrm{img}}\right)+1\cdot \left(W^{\mathrm{tab}⊤}Z^{\mathrm{tab}}\right)=W^{\mathrm{tab}⊤}Z^{\mathrm{tab}}$$

**Null-modality Forward** To formulate a no-input reference, we define the null-modality fusion as:

(13) $$Z_{\mathrm{gpr}}^{⌀}=0\cdot \left(W^{\mathrm{img}⊤}Z^{\mathrm{img}}\right)+0\cdot \left(W^{\mathrm{tab}⊤}Z^{\mathrm{tab}}\right)=0$$

Since Shapley values are typically concerned with relative differences in contribution, and this null case is shared across all Shapley value calculations, it can be safely assigned a fixed value of zero in practice without affecting the consistency of the final attribution results.

Building on this foundation, we adopt a Shapley value framework as a principled attribution method to quantify the degree of modality imbalance in our multimodal regression setting. Given a set of modalities, we define the power set of possible modality combinations as:

(14) $$P=\left\{⌀,\left\{X^{\mathrm{img}}\right\},\left\{X^{\mathrm{tab}}\right\},\left\{X^{\mathrm{img}},X^{\mathrm{tab}}\right\}\right\}$$

For each subset S∈P, we compute a value function v(S) as the negative mean absolute error (MAE) over the validation set when the model is evaluated with only the modalities in *S*:

(15) $$v\left(S\right)=-\mathrm{MAE}\left(Y,{\hat{Y}}^{S}\right)=-\frac{1}{n}\sum_{i=1}^{n}\left|Y_{i}-{\hat{Y}}_{i}^{S}\right|,\;S\in P$$

where ${\hat{Y}}^{S}$ denotes the model prediction with only modality subset *S* available, as defined in our modality ablation forward formulation.

Following the standard two-modality Shapley decomposition, the contribution (or marginal utility) of each modality can be computed as:

(16) $$\begin{array}{cc} \phi^{\mathrm{img}} & =\frac{1}{2}\left[v\left(\left\{X^{\mathrm{img}}\right\}\right)-v\left(⌀\right)\right]+\frac{1}{2}\left[v\left(\left\{X^{\mathrm{img}},X^{\mathrm{tab}}\right\}\right)-v\left(\left\{X^{\mathrm{tab}}\right\}\right)\right]\\ \phi^{\mathrm{tab}} & =\frac{1}{2}\left[v\left(\left\{X^{\mathrm{tab}}\right\}\right)-v\left(⌀\right)\right]+\frac{1}{2}\left[v\left(\left\{X^{\mathrm{img}},X^{\mathrm{tab}}\right\}\right)-v\left(\left\{X^{\mathrm{img}}\right\}\right)\right] \end{array}$$

These values reflect each modality’s average marginal contribution across all possible input subsets. In our implementation, since v(⌀) is a constant term shared across all Shapley computations, it is set to zero without loss of generality. The resulting Shapley scores serve as modality imbalance indicators: lower values indicate weaker modality utility during prediction, highlighting convergence imbalance or representation dominance issues.

Equation (16) provides the formal definition of modality-level Shapley values. In practice, we substitute the contribution function defined in Equation (15) into the expression in Equation (16), yielding the following MAE-based formulation:

(17) $$\begin{array}{cc} \phi^{\mathrm{img}}=\; & \;\frac{1}{2}\left[-\mathrm{MAE}\left(Y,{\hat{Y}}^{\mathrm{img}}\right)+\mathrm{MAE}\left(Y,{\hat{Y}}^{⌀}\right)\right]\\ \; & +\frac{1}{2}\left[-\mathrm{MAE}\left(Y,{\hat{Y}}^{\left(\mathrm{img},\mathrm{tab}\right)}\right)+\mathrm{MAE}\left(Y,{\hat{Y}}^{\mathrm{tab}}\right)\right] \end{array}$$

(18) $$\begin{array}{cc} \phi^{\mathrm{tab}}=\; & \;\frac{1}{2}\left[-\mathrm{MAE}\left(Y,{\hat{Y}}^{\mathrm{tab}}\right)+\mathrm{MAE}\left(Y,{\hat{Y}}^{⌀}\right)\right]\\ \; & +\frac{1}{2}\left[-\mathrm{MAE}\left(Y,{\hat{Y}}^{\left(\mathrm{img},\mathrm{tab}\right)}\right)+\mathrm{MAE}\left(Y,{\hat{Y}}^{\mathrm{img}}\right)\right] \end{array}$$

This formulation allows us to interpret the marginal contribution of each modality as the average reduction in prediction error across all possible inclusion orders.

#### 4.5.2. Adjustment Strategy

Given that the two tasks exhibit distinct modality preferences, we introduce a loss weighting strategy to regulate modality balance during training. Specifically, in the GPR prediction task, the low-dimensional tabular features tend to dominate early convergence due to their high information density. In contrast, the dose difference task, which requires fine-grained spatial matrix regression, relies more heavily on image modality to capture spatial context.

Thus, we propose a dynamic loss weighting strategy based on modality attribution to address modality imbalance. We define the DDP loss coefficient as:

(19) $$\lambda_{\mathrm{DDP}}=1+\max\left(\phi^{\mathrm{tab}}-\phi^{\mathrm{img}},0\right)\cdot r^{2}$$

where $\phi^{\mathrm{tab}}$ is the Shapley value quantifying the contribution of the tabular modality in GPR prediction. *r* is the **Modality Balance Factor** as the hyperparameter. The overall training objective is thus formulated as:

(20) $$L=L_{\mathrm{gpr}}+\lambda_{\mathrm{DDP}}\cdot L_{\mathrm{ddp}}$$

This formulation increases the emphasis on the image modality (via the DDP task) when the tabular modality exhibits dominance, helping to balance modality convergence across tasks.

Algorithm 1 shows the per-epoch training procedure of **BMMQA**.
**Algorithm 1:** Per-epoch Training Procedure of **BMMQA**
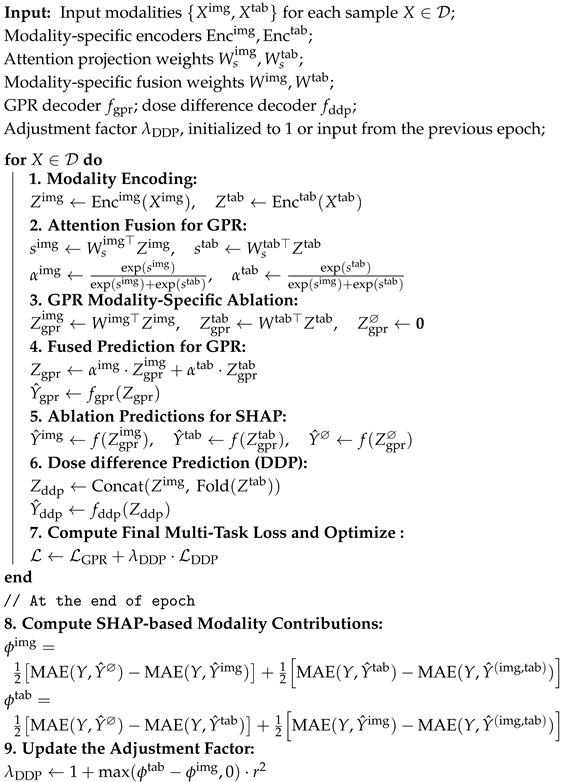


## 5. Evaluation and Results

This section comprehensively analyzes our proposed **BMMQA** for achieving robust automated PSQA.

### 5.1. Implementation Setting

The framework processes 512×512 resized dose arrays through a dual-task architecture: (1) a GPR regression head (AdaptiveAvgPool2d → Flatten → Dropout(0.1) → Linear(512, 3)) predicting three gamma criteria, and (2) a U-Net decoder with channels [768, 384, 192, 128, 32] for dose prediction. Training used Adam ($\eta =5\times 10^{-4}$) with a 32-batch/30-epoch configuration and a 7:1:2 Train:Validation:Test split, with regularization applied via dropout (p=0.1) throughout the network as detailed in Table 5. To ensure representativeness, we employed multi-factor stratified sampling (Figure 2), taking into account both binned Gamma Passing Rates (continuous regression targets) and lesion categories (categorical feature). This approach prevents biased splits (e.g., training only on success cases or rare lesion types in isolation) and ensures that each subset covers the full difficulty spectrum and lesion diversity, thereby improving fairness and reproducibility.

### 5.2. Evaluation Metrics

We evaluate **BMMQA** performance using mean absolute error (MAE), Root Mean Square Error (RMSE) and $R^{2}$ for the GPR task, and both MAE and Structural Similarity Index Measure (SSIM) for the DDP task.

(21) $$\mathrm{MAE}=\frac{1}{n}\sum_{i=1}^{n}\left|y_{i}-{\hat{y}}_{i}\right|$$

(22) $$\mathrm{RMSE}=\sqrt{\frac{1}{n}\sum_{i=1}^{n}{\left(y_{i}-{\hat{y}}_{i}\right)}^{2}}$$

(23) $$R^{2}=1-\frac{\sum_{i=1}^{n}{\left(y_{i}-{\hat{y}}_{i}\right)}^{2}}{\sum_{i=1}^{n}{\left(y_{i}-\bar{y}\right)}^{2}}$$

(24) $$\mathrm{SSIM}\left(X_{1},X_{2}\right)=\frac{\left(2\mu_{X_{1}}\mu_{X_{2}}+C_{1}\right)\left(2\sigma_{X_{1}X_{2}}+C_{2}\right)}{\left(\mu_{X_{1}}^{2}+\mu_{X_{2}}^{2}+C_{1}\right)\left(\sigma_{X_{1}}^{2}+\sigma_{X_{2}}^{2}+C_{2}\right)}$$

### 5.3. Baseline Methods

This subsection presents the baseline PSQA methods as benchmarks for comparison with the proposed **BMMQA** approach.

Previous studies have predominantly focused on single-modal approaches, which form the foundation of the single-modal baseline methods selected in this work.

To comprehensively evaluate the impact of different modalities in PSQA, we categorized our experimental methods into tabular, image, and unbalanced (a combination of tabular and image modalities) and balanced multimodalities. For each setting, both tasks (GPR and DDP) were trained simultaneously. We then analyzed the performance across various metrics and visualized the results using diagrams and tables.

Methods based on the tabular modality have primarily relied on traditional machine learning approaches in previous studies. In particular, regression tree models and linear regression models have been the most widely adopted in PSQA research: tree-based models have been employed in several works [41,49,50,51], while linear regression models have also been tested in related studies [41,50,52], including Poisson Lasso regression as a representative approach. Following Zhu et al. (2023) [41], we therefore employed **Gradient Boosting Decision Tree (GBDT)**, **Random Forest (RF)**, and **Poisson Lasso (PL)** models as representative baselines. These models utilize manually designed complexity metrics as input features to predict the Gamma Passing Rate (GPR), ensuring consistency with prior work, enabling direct comparability, and supporting reproducibility. Detailed descriptions of their implementation can be found in [41].

For image modality baselines, ref. [53] proposed a ResNet-based **UNet++** architecture for predicting GPR and dose differences, while [54] introduced **TransQA**, a Transformer-based model designed for the same tasks. To address data imbalance, ref. [8] combined **RankLoss** with a DenseNet backbone to enhance the robustness of GPR predictions. For consistency in comparison, we also incorporated **RegNet** [55] to evaluate the performance of a more recent CNN backbone.

In our study, the last two categories of methods—unbalanced and balanced multimodal approaches—share the same network architecture, with the key distinction being whether a modality balancing algorithm is applied. Since the tabular modality is relatively simple, it typically employs a fixed encoder structure [10]; therefore, the main variation across settings lies in the choice of the image modality encoder. Specifically, we selected **ResNet18** [53,56], **DenseNet121** [8,15,57], **MobileVit** [54,58], and **RegNet** [55,59]. ResNet and DenseNet represent widely adopted classical CNN backbones in prior PSQA-related studies, MobileVit provides a lightweight variant, while RegNet is a relatively recent CNN family proposed around the same period as Transformer-based approaches and has demonstrated promising potential in medical imaging applications. Together, these encoders cover classical, lightweight, and modern high-performing architectures, ensuring consistency with prior baselines and enabling fair comparisons.

The main architectures employed in PSQA prediction are convolutional neural networks (CNNs) and, more recently, Transformer-based models. Among CNNs, ResNet and DenseNet have been widely adopted in prior PSQA-related studies [8,15,53,56,60], while MobileVit [54,58] represents a lightweight variant. To complement these, we also included RegNet [61], a relatively recent CNN family proposed around the same period as Transformer-based approaches, which has demonstrated promising potential in medical image generation tasks [59]. Together, these encoders provide a representative spectrum across classical, lightweight, and modern high-performing architectures, ensuring consistency with prior baselines and enabling fair comparisons.

The overall experimental settings remain largely consistent for the DDP task. However, since the tabular modality alone cannot independently accomplish the DDP task, it is not included in the corresponding results table.

### 5.4. EXP-A: Comparative Experiment with PSQA Methods

#### 5.4.1. GPR Result Analysis

Table 6 reports the GPR prediction performance of various models under three commonly used gamma criteria (1%/1 mm, 2%/2 mm, and 2%/3 mm), with results further stratified by ground truth GPR intervals: All, 95–100, 90–95, and <90. The choice of intervals follows the recommendations of the AAPM Task Group 218 report [34], which defines a **universal tolerance limit** of GPR ≥ 95% (with 3%/2mm criteria and 10% dose threshold) for clinical acceptance, and a **universal action limit** of GPR ≥ 90% under the same criteria. Therefore, we stratified the results into three clinically meaningful categories: 95–100% (passing and acceptable), 90–95% (borderline, close to the action limit), and <90% (failing plans requiring further investigation). Since $R^{2}$ is known to be overly sensitive under sparse subgroup data distributions, we only report the overall (All) $R^{2}$ values to ensure meaningful and stable interpretation.

This stratification allows for a more nuanced evaluation beyond global accuracy, especially regarding clinically relevant but underrepresented failure cases. In practical radiotherapy quality assurance, plans with GPR < 90% are usually considered to have failed. Misclassifying these failing plans as acceptable poses a significant clinical risk, far greater than conservative rejection of marginally acceptable plans. Moreover, such failure cases account for only a small proportion of the dataset, making the <90 subgroup rare and highly valuable for evaluating model robustness and practical deployment readiness.

**Figure 5:**
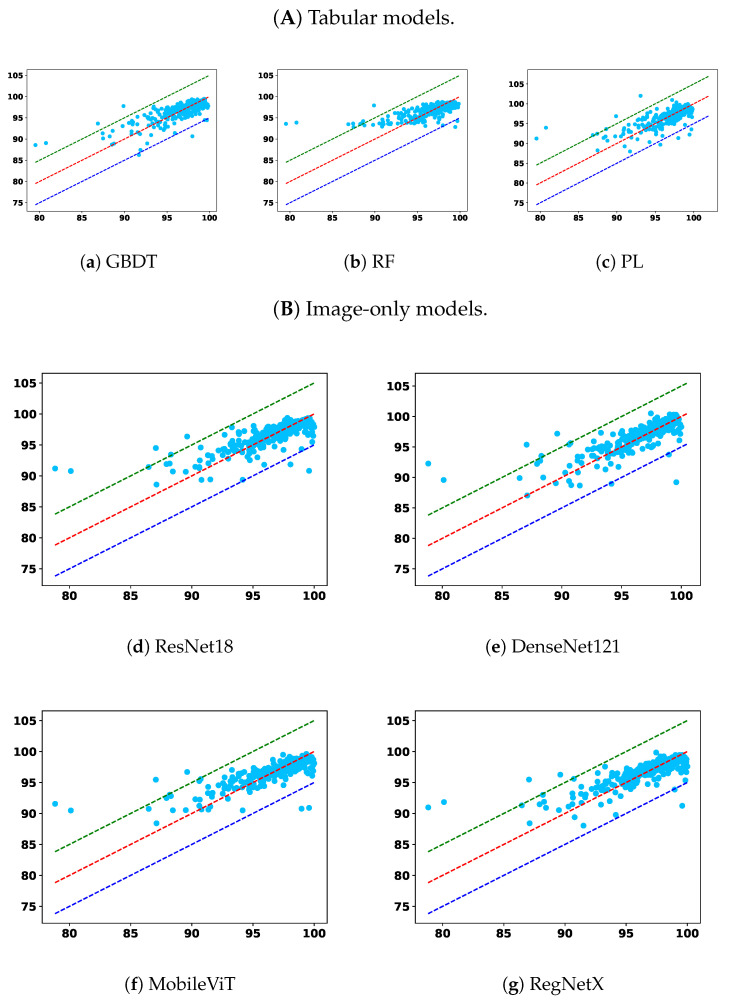
Comparison across the remaining two model types: (**A**) tabular only and (**B**) image only.

**Figure 6:**
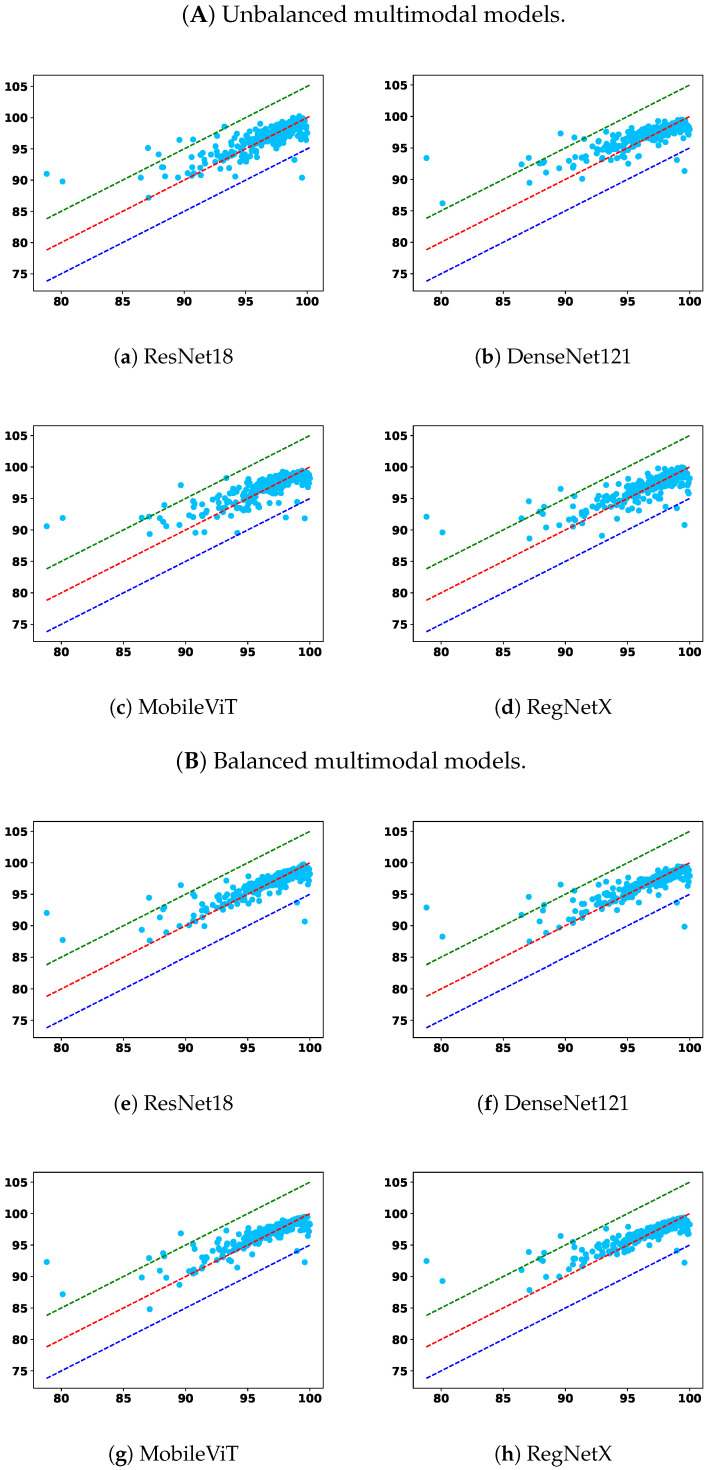
Comparison across the remaining two model types: (**A**) unbalanced multimodal and (**B**) balanced multimodal.

To complement the tabulated results, Figure 5 and Figure 6 show GPR scatter plots under the 2%/2mm criterion, with ground-truth GPR on the x-axis and predicted GPR on the y-axis. Light-blue dots represent individual samples. The diagonal dashed lines indicate prediction bands: the red line denotes the top 5% correct predictions, the green line the upper 5% deviation, and the dark-blue line the lower 5% deviation.

From the overall trend, it is clear that models trained solely on the tabular modality underperform across nearly all settings. Among them, RF and PL show lower overall accuracy and higher prediction errors in the <90 subgroup, indicating poor generalization and limited robustness. While the GBDT model demonstrates relatively better robustness in identifying failure cases under relaxed criteria, namely, 2%/2 mm and 2%/3 mm, this comes at the cost of diminished performance in the 95–100 and overall categories, suggesting an overfitting tendency toward rare cases or a lack of adaptability across GPR ranges. This reflects a broader limitation of relying on low-dimensional tabular features alone: such features may capture plan complexity but lack the spatial context necessary for comprehensive dose distribution assessment.

Image-only models, on the other hand, benefit from richer spatial information and demonstrate superior overall accuracy across nearly all criteria. Notably, models such as UNet++, TransQA, and RegNet consistently outperform tabular baselines in the “All” and 90–100 interval. However, their performance in the critical <90 subgroup is not always optimal. For instance, in some settings, the RMSEs in this subgroup are higher than those of GBDT, indicating a potential weakness in detecting outliers or failure cases. This suggests that while image modality provides strong general pattern learning capabilities, it may require additional mechanisms to capture minority failure modes effectively.

Multimodal models integrate image and tabular information and offer a compelling balance between overall performance and robustness. Across all settings, multimodal models outperform their unimodal counterparts, confirming the complementary nature of these modalities. More importantly, the proposed modality-balanced variants demonstrate consistent improvements over unbalanced versions. This is particularly evident in the <90 subgroup, where balanced models reduce the MAE and RMSE for nearly all encoder types. The benefit is pronounced in ResNet18, DenseNet121, and MobileViT-based variants, where balancing helps prevent the tabular modality from prematurely dominating the learning process. This validates our hypothesis that early convergence of simpler modalities can hinder effective feature fusion, and that regulating this imbalance can substantially enhance the model’s ability to improve overall prediction fidelity and remain sensitive to critical failure cases.

Although RegNet-based balanced models show slightly less consistent improvements—especially in RMSE under strict criteria—their performance remains competitive and still offers benefits in terms of MAE. This variation may stem from architectural sensitivity or optimization dynamics, suggesting that the effect of balancing may interact with encoder complexity.

These findings highlight the necessity of addressing modality imbalance in multimodal learning for PSQA. Simple fusion of modalities without considering training dynamics can lead to suboptimal utilization of complementary information. Our approach, which combines learning rate modulation and gradient normalization, offers an effective solution. In particular, the observed robustness improvements in the <90 subgroup emphasize the clinical importance of balanced learning for enhancing average accuracy and ensuring patient safety by reducing the likelihood of high-risk misclassifications. As such, modality balancing should be considered a key component in building safe, trustworthy AI systems for clinical deployment in radiotherapy quality assurance.

In addition to MAE and RMSE, the $R^{2}$ values in Table 6 further corroborate these observations. Tabular-only models generally yield a lower $R^{2}$, often below 0.60, confirming their limited explanatory power and inability to capture the variance in GPR outcomes. Image-only models achieve moderately higher $R^{2}$ (around 0.62–0.64), reflecting their stronger alignment with ground-truth variations but with persistent weaknesses in the <90 subgroup. In contrast, multimodal models—particularly the balanced variants—consistently attain the highest $R^{2}$ values across nearly all criteria, with RegNet-based models exceeding 0.67 in some settings. These improvements indicate not only reduced absolute errors but also enhanced consistency in capturing the underlying variance structure of GPR. Importantly, the higher $R^{2}$ in the balanced multimodal models suggests improved generalization, supporting their reliability for both routine QA and the detection of clinically critical outliers.

#### 5.4.2. DDP Result Analysis

Table 7 reports the DDP performance under different modality configurations and encoder backbones. We evaluate MAE (%), SSIM and $R^{2}$, with further stratification by ground truth GPR intervals: 95–100, 90–95, and <90, in addition to the overall average. These strata correspond to varying clinical acceptance levels, with lower GPR values indicating more challenging cases. Since $R^{2}$ is highly sensitive to small sample sizes and may yield unstable or even misleading values when computed on narrow strata, we report $R^{2}$ values only for the overall (all samples) case to ensure robustness and interpretability.

Since the tabular modality lacks spatial resolution, we focus on three representative settings: (1) image-only baselines, (2) unbalanced multimodality (MM) models without contribution control, and (3) balanced multimodality (BMM) models using our proposed modality regulation mechanism.

From Table 7, we observe that unbalanced fusion can degrade DDP performance, sometimes even underperforming image-only models—especially in high-GPR regions. This suggests that DDP is primarily image-sensitive, and that naive modality fusion can introduce interference when less-suitable modalities dominate. In contrast, BMM models consistently achieve the lowest MAE, highest SSIM, and strongest $R^{2}$ values across backbones, highlighting the value of modality balancing in spatially structured prediction. The consistently higher $R^{2}$ further indicates that BMM models explain a greater proportion of variance in voxel-wise dose differences, reinforcing their predictive robustness.

**Figure 7:**
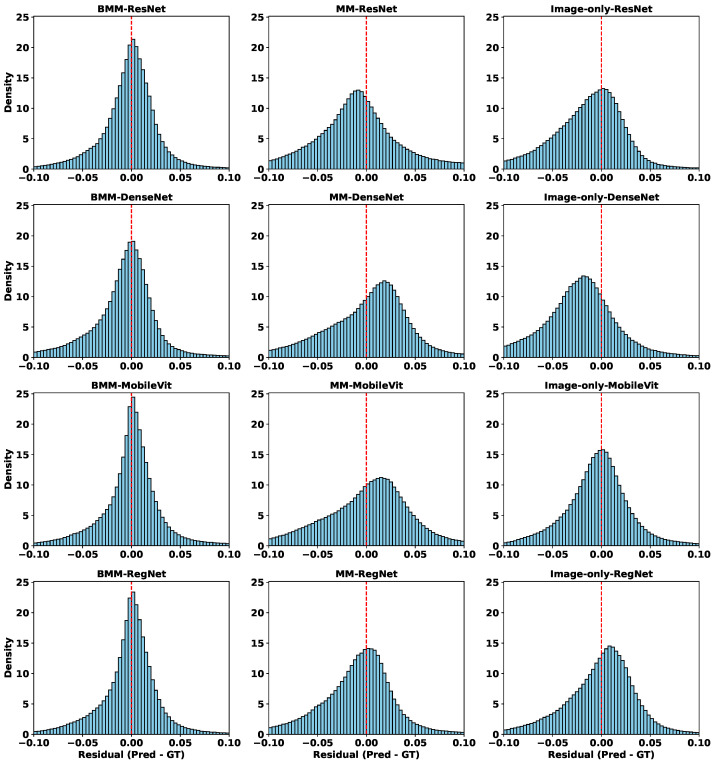
Residual distributions of different models in the DDP task. Each row corresponds to a different backbone (ResNet, DenseNet, MobileViT, RegNet), and each column represents a different modality configuration: balanced multimodal (BMM), unbalanced multimodal (MM), and image-only.

To better interpret these quantitative results, Figure 7 shows residual distributions across all modality-backbone combinations. Each row corresponds to a backbone (ResNet, DenseNet, MobileViT, RegNet), and columns represent BMM, MM, and image-only settings. The histograms confirm that BMM models not only reduce average errors, but also produce more symmetric, narrow residual distributions centered near zero—indicating better generalization and reduced voxel-level variance. In contrast, MM models show broader and often skewed distributions, reflecting prediction instability due to unregulated modality mixing.

**Figure 8:**
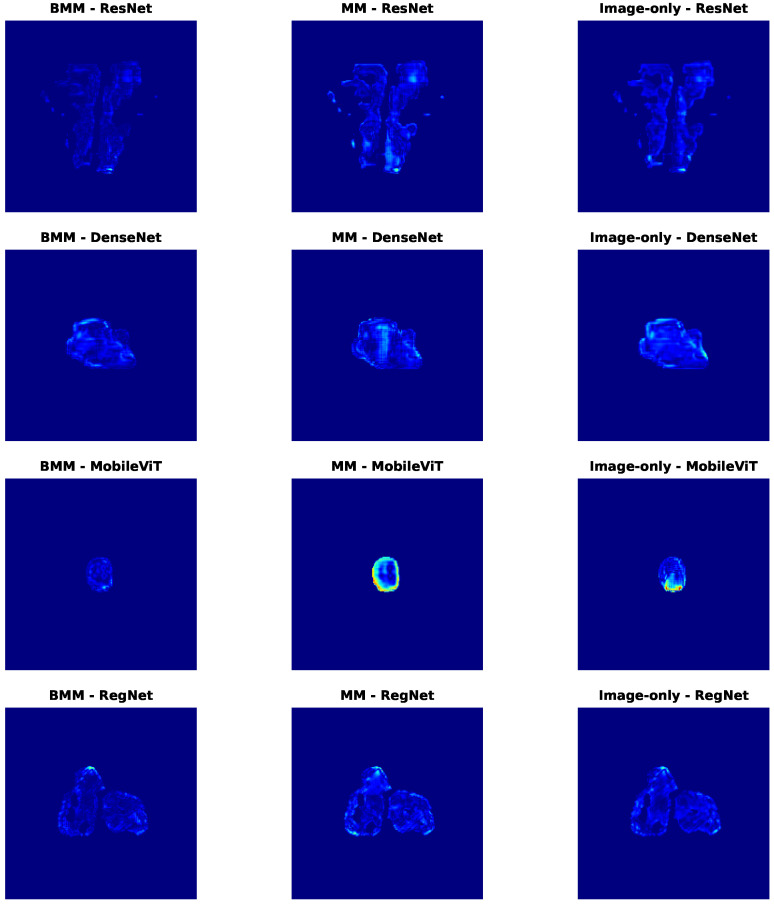
Dose difference error maps for selected samples in the DDP task. Each row corresponds to a different backbone (ResNet, DenseNet, MobileViT, RegNet), and each column represents a different modality configuration: balanced multimodal (BMM), unbalanced multimodal (MM), and image-only. Darker red areas indicate larger differences, whereas lighter blue areas indicate smaller differences.

To further explore spatial behavior, Figure 8 visualizes voxel-wise error maps on selected test cases. Consistently, BMM models yield cleaner and more localized error patterns. Notably, MobileViT-MM exhibits strong overestimation artefacts (bright yellow) in the center, which are suppressed mainly in its BMM counterpart. Similar spatial refinements are observed across other backbones, especially in clinically critical regions.

Together, these results demonstrate that modality regulation not only improves quantitative metrics like MAE and SSIM, but also enhances spatial fidelity—reducing high-error artifacts and increasing clinical reliability. The complementary insights from Table 7 and Figure 7 and Figure 8 strongly validate the effectiveness of our balanced multimodal strategy in spatially sensitive PSQA tasks.

#### 5.4.3. Modality Contribution Dynamics During Training

**Figure 9:**
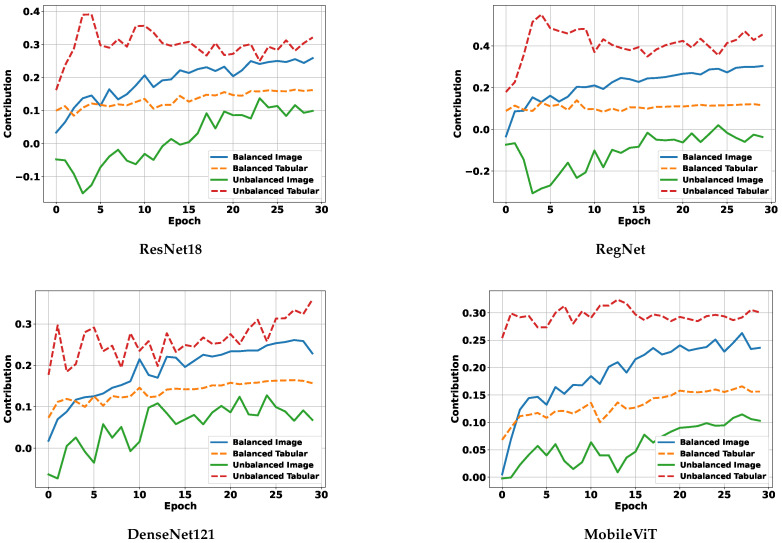
Modality contribution curves for different backbones.

Figure 9 presents the evolution of modality contributions across training epochs for four representative backbone encoders. In the unbalanced setting (dashed lines), the tabular modality consistently dominates, exhibiting significantly higher contribution scores than the image modality across all backbones. This imbalance persists throughout training and suggests that the network overfits to the easier-to-learn tabular features while underutilizing spatial information, which is critical for DDP.

In contrast, under our balanced multimodality strategy (solid lines), modality contributions become more equitable. The image modality sees a clear increase in contribution, while the tabular modality’s influence is moderated, resulting in a more stable and task-appropriate fusion. These results empirically demonstrate that our balancing mechanism effectively mitigates modality dominance during joint training.

When viewed alongside Table 7, it is evident that balanced modality contributions lead to superior DDP performance, especially for image-sensitive tasks. This further validates our claim that addressing modality imbalance is essential for achieving both accurate and robust predictions in multimodal PSQA models.

### 5.5. EXP-B: Ablation Experiment with Modality Balance Factor *r*

We conduct an ablation experiment to analyze the influence of the hyperparameter *r* in the formulation of $\lambda_{\mathrm{DDP}}$. This parameter serves as an amplification factor, dynamically rebalancing the contributions of different modalities, particularly when one modality dominates the learning process. In this experiment, for each fixed *r*, we train a separate model and record its final DDP prediction error (MAE) under three standard GPR thresholds.

**Figure 10:**
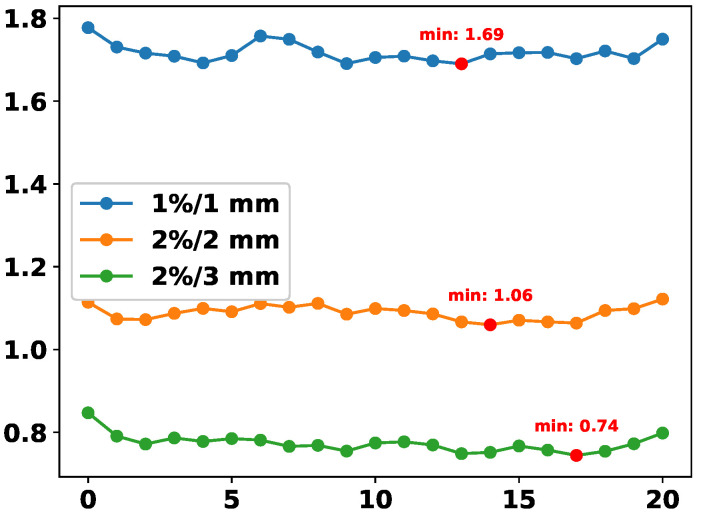
DDP error (MAE) across different values of *r* in the computation of $\lambda_{\mathrm{DDP}}$. Each curve corresponds to a different GPR threshold.

As shown in Figure 10, the error curves generally decrease as *r* increases, reaching their minima around r=14 to r=17 for all three criteria. This indicates that moderate amplification of modality imbalance yields the best DDP accuracy. When *r* is too small (e.g., close to 0), modality imbalance is insufficiently addressed, while overly large values (e.g., r>18) may lead to overcompensation and degrade performance. The consistent minima across criteria demonstrate the robustness of our balancing strategy.

### 5.6. Computational Resource Utilization

SHAP-based models are often associated with concerns regarding computational cost, as exact SHAP computation typically requires exponential complexity. However, in our proposed framework, we circumvent this limitation by enabling the simultaneous derivation of multiple modality ablation results within a single forward pass, thus significantly reducing the overhead traditionally linked to SHAP.

Table 8 compares the computational resource usage of our **BMMQA** framework against several deep learning-based PSQA baselines. The comparison includes model names, floating-point operations (FLOPs in GMac), and parameter counts (in millions). Among the baselines, Unet++, TranQA, and RegNet represent PSQA models with varying computational demands.

Notably, **BMMQA** achieves competitive performance with substantially lower FLOPs and parameter size, underscoring its efficiency and suitability for resource-constrained or computation-sensitive clinical scenarios.

## 6. Conclusions

Through a comprehensive review and empirical analysis of existing multimodal deep learning approaches for PSQA, we identified modality imbalance—where faster-converging tabular features dominate training and suppress image feature learning—as a key factor limiting model robustness and clinical reliability. To address this, we propose **BMMQA**, a modality- and task-aware multimodal framework that explicitly regulates modality contributions during training. **BMMQA** leverages SHAP-derived imbalance indicators with task-specific weighting strategies to balance convergence across modalities, and incorporates a fast network forward mechanism along with tailored fusion protocols—attention-based weighting for GPR and spatial concatenation for DDP—to enhance feature collaboration. Experiments on a large clinical IMRT dataset show that **BMMQA** consistently outperforms state-of-the-art fusion baselines in both coarse-grained (GPR) and fine-grained (DDP) tasks, while improving interpretability and reliability. These results highlight the importance of explicitly addressing modality imbalance for the safe and effective deployment of AI in radiotherapy quality assurance, establishing a novel example in imbalanced multi-task multimodal learning.

This study has two main limitations. First, the dataset was collected from a single center (PUMCH) within a limited timeframe, which may restrict the generalizability of the results and introduce potential biases associated with technological evolution. Second, the current framework relies on only two modalities: image-based dose distributions and tabular plan complexity features, while excluding other potentially informative clinical data, such as CT scans or electronic health records (EHRs). Future work will aim to validate the framework on multi-center, multi-device datasets to enhance robustness and to extend the multimodal design by incorporating additional clinical information, thereby further improving predictive performance and clinical applicability.

## Figures and Tables

**Table 1:** Summary of multimodal PSQA methods. Symbols: ✓ = Yes; ✗ = No.

Reference	Category	Description (Modalities + Fusion Strategy)	Fine-Grained Task?	Modality Balance?
[11]	Early Fusion	Dose matrices encoded via DenseNet-121 (image) and plan complexity features (tabular) are separately reduced using PCA/Lasso and concatenated for Random Forest regression.	✗	✗
[13]	Early Fusion	Plan and dosimetric features (tabular) are selected via Lasso and used as input to a logistic regression model for classification.	✗	✗
[14]	Late Fusion	Two SVMs are trained separately on radiomics (image-like features) and plan complexity features; predictions are aggregated to improve GPR classification and regression.	✗	✓
[10]	Late Fusion	Dose distribution matrices (image) and MU values (tabular) are processed by separate models; outputs are aggregated at the decision level as a baseline fusion strategy.	✗	✓
[10]	Intermediate Fusion	3D ResNet encodes MLC aperture images; spatial features are concatenated one-to-one with MU values (tabular), jointly optimized in an end-to-end model (FDF).	✓	✗
[12]	Intermediate Fusion	Dose distribution is compressed into a 99D vector and concatenated with MU value; the fused vector is passed to fully connected layers.	✓	✗
[15]	Intermediate Fusion	MU-weighted fluence maps generated from delivery log files are used as image input to DenseNet for GPR prediction under multiple gamma criteria.	✓	✗
BMMQA	Intermediate Fusion	Dual-modality input (dose matrix + tabular metrics); image encoded by a pre-trained CNN, tabular by MLP; fusion uses task-specific heads: attention-weighted feature fusion for GPR, spatial concatenation for DDP; Shapley-based diagnostics and adaptive loss scaling address modality imbalance.	✓	✓

**Table 2:** Treatment plans by site.

Treatment Site	Abbreviation	Number of Plans
Head and Neck	H&N	19
Chest	C	138
Abdomen	A	31
Pelvis	P	22

**Table 3:** Selected complexity metrics, their references, and one sample in each lesion, namely, Head and Neck (H&N), Chest (C), Abdominal (A), and Pelvic (P).

Type	Ref	Metric Name	H&N	C	A	P
Complexity Metric (Input Tabular Modality)	[35]	Aperture area variability of distal MLC (d-AAV)	0.30	0.42	0.35	0.43
		Aperture area variability of proximal MLC (p-AAV)	0.31	0.44	0.41	0.43
		Leaf sequence variability of distal MLC (d-LSV)	2.49	3.17	6.81	4.71
		Leaf sequence variability of proximal MLC (p-LSV)	1.44	1.66	3.48	1.88
		Modulation complex score of distal MLC (d-MCS)	1.38	0.90	0.97	0.82
		Modulation complex score of proximal MLC (p-MCS)	1.21	0.70	0.68	0.73
	[36]	Beam area of distal MLC (d-BA)	0.27	0.56	0.34	0.38
		Beam area of proximal MLC (p-BA)	0.22	0.53	0.37	0.35
		Union aperture areas of distal MLC (d-UAA)	20.45	22.84	16.89	35.90
		Union aperture areas of proximal MLC (p-UAA)	15.43	20.45	18.44	32.26
		Plan irregularity of distal MLC (d-PI)	32.98	66.99	88.17	66.18
		Plan irregularity of proximal MLC (p-PI)	31.57	66.84	92.33	68.85
		Plan modulation of distal MLC (d-PM)	0.70	0.58	0.65	0.57
		Plan modulation of proximal MLC (p-PM)	0.69	0.56	0.59	0.57
Complexity Metric (Input Tabular Modality)	[37]	Circumference/area of distal MLC (d-C/A)	0.36	1.01	1.12	1.00
		Circumference/area of proximal MLC (p-C/A)	0.34	1.02	1.32	1.01
	[38]	Closed leaf score of distal MLC (d-CLS)	0.65	1.07	1.79	1.54
		Closed leaf score of proximal MLC (p-CLS)	0.66	1.08	2.11	1.55
		Mean asymmetry distance of distal MLC (d-MAD)	0.78	0.79	0.78	0.80
		Mean asymmetry distance of proximal MLC (p-MAD)	0.77	0.81	0.79	0.79
	[39]	Small aperture score 2 mm of distal MLC (d-SAS-2 mm)	0.23	0.34	0.26	0.34
		Small aperture score 5 mm of distal MLC (d-SAS-5 mm)	0.24	0.36	0.31	0.34
		Small aperture score 10 mm of distal MLC (d-SAS-10 mm)	263.53	195.03	191.07	172.01
		Small aperture score 15 mm of distal MLC (d-SAS-15 mm)	10.01	28.43	31.07	28.29
		Small aperture score 2 mm of proximal MLC (p-SAS-2 mm)	9.87	29.62	38.17	29.59
		Small aperture score 5 mm of proximal MLC (p-SAS-5 mm)	0.19	0.15	0.11	0.13
		Small aperture score 10 mm of proximal MLC (p-SAS-10 mm)	0.10	0.08	0.11	0.08
		Small aperture score 15 mm of proximal MLC (p-SAS-15 mm)	0.26	0.19	0.13	0.17
	[40]	Average leaf gap of distal MLC (d-ALG)	0.16	0.14	0.15	0.13
		Average leaf gap of proximal MLC (p-ALG)	0.49	0.37	0.23	0.25
		Standard deviation of leaf gap of distal MLC (d-SLG)	0.31	0.33	0.20	0.22
		Standard deviation of leaf gap of proximal MLC (p-SLG)	0.68	0.53	0.31	0.39
	[41]	MU value of each beam (MU)	0.59	0.48	0.31	0.37
QA Metric (Target)		Gamma Passing Rate (1%/1mm)	91.80	93.72	99.15	97.90
		Gamma Passing Rate (2%/2mm)	96.58	96.78	99.78	99.05
		Gamma Passing Rate (3%/2mm)	98.31	98.13	99.95	99.37

**Table 4:** Mathematical symbol table.

Symbol	Description
$X=\{X^{\mathrm{img}},X^{\mathrm{tab}}\}$	A single input sample, consisting of an image modality $X^{\mathrm{img}}\in R^{H\times W}$ and a tabular modality $X^{\mathrm{tab}}\in R^{33}$.
$Y_{\mathrm{gpr}}$, $Y_{\mathrm{ddp}}$	Ground truths for GPR and DDP tasks, respectively. $Y_{\mathrm{gpr}}\in R^{3}$; $Y_{\mathrm{ddp}}\in R^{H\times W}$.
$Z^{\mathrm{img}}$	Latent feature map from the image encoder; shape: $N_{c}\times H_{z}\times W_{z}$. Requires pooling before projection.
$Z^{\mathrm{tab}}$	Latent tabular embedding vector produced by the tabular encoder; shape: $N_{t}$.
$Z_{\mathrm{gpr}},\;Z_{\mathrm{ddp}}$	Fused latent representations used in GPR and DDP heads, respectively.
$W^{⊤}Z^{\mathrm{tab}}$	Matrix multiplication between projection matrix and tabular embedding vector.
$W^{⊤}Z^{\mathrm{img}}$	Matrix multiplication between the projection matrix and the pooled image feature map. Due to the complexity introduced by both multi-task and multimodal notation, spatial pooling over $Z^{\mathrm{img}}\in R^{N_{c}\times H\times W}$ is applied implicitly for notational simplicity and consistency, resulting in an $N_{c}$-dimensional vector. This convention is used throughout the paper, including expressions such as $W_{s}^{\mathrm{img}⊤}Z^{\mathrm{img}}$.
$\alpha^{\mathrm{img}},\;\alpha^{\mathrm{tab}}$	Attention weights for the image and tabular modalities, computed via softmax over scalar scores.
P	Power set of modality subsets considered for ablation: $\left\{⌀,\left\{X^{\mathrm{img}}\right\},\left\{X^{\mathrm{tab}}\right\},\left\{X^{\mathrm{img}},X^{\mathrm{tab}}\right\}\right\}$.
${\hat{Y}}_{\mathrm{gpr}}^{S}$	GPR prediction computed using only the modality subset S∈P.
${\hat{Y}}_{\mathrm{gpr}},\;{\hat{Y}}_{\mathrm{ddp}}$	Model predictions for GPR and DDP tasks using all available modalities by default, namely ${\hat{Y}}_{\mathrm{gpr}}={\hat{Y}}_{\mathrm{gpr}}^{\left(\mathrm{img},\mathrm{tab}\right)}\in R^{3}$; ${\hat{Y}}_{\mathrm{ddp}}={\hat{Y}}_{\mathrm{ddp}}^{\left(\mathrm{img},\mathrm{tab}\right)}\in R^{H\times W}$.
v(S)	Value function for modality subset S⊆P, defined as the negative MAE of predictions ${\hat{Y}}^{S}$ over the validation set. Used to compute Shapley-style contributions.
$\phi^{\mathrm{img}},\;\phi^{\mathrm{tab}}$	Modality contributions computed via two-modality Shapley decomposition based on v(S).
$\lambda_{\mathrm{ddp}}$	Adaptive weighting factor for the DDP loss, derived from modality contributions.
$L_{\mathrm{gpr}},\;L_{\mathrm{ddp}}$	Loss terms for the GPR and DDP tasks.
L	Total training loss: $L=L_{\mathrm{gpr}}+\lambda_{\mathrm{ddp}}\cdot L_{\mathrm{ddp}}$.

**Table 5:** Parameter settings.

Parameter	Description	Value
Resized Dose Plan Array Shape	To organize dose plan array samples into a batch, we perform ResizeWithPadOrCrop preprocessing	[512, 512]
Regression Head Architecture	Architecture of GPR prediction output; the output dimension is 3 since we select three GPR criteria [1%/1 mm, 2%/2 mm, 2%/3 mm]	AdaptiveAvgPool2d[1, 1] Flatten() Dropout(*p* = 0.1) Linear[512, 3]
Decoder (DDP) Architecture	U-Net decoder channel dimensions	[768, 384, 192, 128, 32]
Dropout Rate	The dropout rate employed in network	0.1
Optimizer	Type of optimizer used	Adam
r	The hyperparameters used in the computation of $\lambda_{\mathrm{ddp}}$ are set to their default values unless otherwise specified	16
Epochs	Number of training epochs	30
Batch Size	Size of the training batches	32
Learning Rate	Learning rate for the optimizer	$5\times 10^{-4}$
Dataset Split Ratio (EXP-A and EXP-B)	Train:Validation:Test split ratio	Multi-factor stratified sampling based on Figure 2, with a 7:1:2 split.

**Table 6:** GPR performance comparison.

Category	Method	Criteria	GPR MAE (%)				GPR RMSE (%)				$R^{2}$
			All	95–100	90–95	<90	All	95–100	90–95	<90	
Tabular Modality	GBDT [41]	1%/1 mm	1.8666	1.6246	1.4805	3.3034	2.6750	2.3497	1.9099	4.3705	0.6055
		2%/2 mm	1.2371	0.9154	1.9578	4.3461	1.8317	1.2805	2.3500	5.2914	0.5992
		2%/3 mm	0.8798	0.7188	2.2047	7.8881	1.3434	1.0064	2.6306	7.8911	0.5740
	RF [41]	1%/1 mm	2.0911	1.7536	1.5255	4.1621	3.0146	2.2812	1.8969	5.5119	0.5305
		2%/2 mm	1.3554	0.9389	1.9412	7.0523	2.1110	1.2655	2.2275	7.7729	0.5030
		2%/3 mm	0.9515	0.6937	3.0804	10.4988	1.5628	0.9491	3.2777	10.5093	0.4784
	PL [41]	1%/1 mm	2.0605	1.6859	1.7921	3.4642	3.1027	2.4075	2.6953	4.7986	0.4821
		2%/2 mm	1.3673	1.0289	1.9915	5.2811	2.1353	1.4706	2.6279	6.5024	0.4697
		2%/3 mm	0.9664	0.7833	2.2965	9.8307	1.5707	1.1072	2.9172	9.8389	0.4349
Image Modality	UNET++ [53]	1%/1 mm	1.7783	1.3875	1.5326	3.1709	2.7271	2.2103	2.0726	4.4958	0.6165
		2%/2 mm	1.1216	0.8269	1.4684	5.4498	1.8683	1.2849	1.8351	6.4454	0.6218
		2%/3 mm	0.7704	0.6126	1.8108	9.2442	1.3504	0.9324	2.2840	9.2930	0.6258
	Ranking Loss [8]	1%/1 mm	1.7604	1.2448	1.4286	3.6156	2.7392	1.8543	1.9004	5.0189	0.6286
		2%/2 mm	1.1131	0.8030	1.5527	5.3085	1.8941	1.2502	1.9655	6.5941	0.6157
		2%/3 mm	0.7512	0.5939	1.7643	9.4602	1.3703	0.8979	2.4679	9.6021	0.6129
	TransQA [62]	1%/1 mm	1.7308	1.4348	1.3519	3.2477	2.6730	2.3100	1.7716	4.5237	0.6344
		2%/2 mm	1.1060	0.7795	1.5627	5.5561	1.8772	1.2354	1.9102	6.6028	0.6297
		2%/3 mm	0.7787	0.6049	1.9731	9.5395	1.3874	0.9178	2.5051	9.5951	0.6171
	RegNet [61]	1%/1 mm	1.7576	1.5107	1.2643	3.4472	2.7098	2.1745	1.6929	4.8423	0.6302
		2%/2 mm	1.0729	0.7354	1.5806	5.4991	1.8329	1.1409	1.9207	6.6090	0.6423
		2%/3 mm	0.7232	0.5699	1.6869	9.4960	1.3191	0.8856	2.1924	9.4994	0.638
Unbalanced Multimodality	ResNet18 [63]	1%/1 mm	1.7590	1.2781	1.5838	3.1678	2.6695	1.9935	2.0368	4.5546	0.6435
		2%/2 mm	1.0841	0.8023	1.4118	5.2429	1.8383	1.2270	1.9120	6.3409	0.6371
		2%/3 mm	0.7587	0.5900	1.9359	9.0621	1.3604	0.9105	2.5084	9.1153	0.6204
	DenseNet121 [53]	1%/1 mm	1.7163	1.4312	1.2647	3.3849	2.5983	2.0865	1.6889	4.5853	0.6701
		2%/2 mm	1.0943	0.7143	1.7679	5.5857	1.8488	1.1296	2.1092	6.4863	0.6681
		2%/3 mm	0.7978	0.6084	2.2694	8.4039	1.3743	0.9064	2.6525	8.9947	0.6399
	MobileVit [62]	1%/1 mm	1.7383	1.3995	1.4173	3.2042	2.6178	2.0552	1.8679	4.5078	0.6577
		2%/2 mm	1.1021	0.7656	1.6221	5.4503	1.8338	1.1682	1.9683	6.4490	0.6443
		2%/3 mm	0.7666	0.5865	2.0116	9.7678	1.3761	0.8995	2.4443	9.7681	0.6208
	RegNet [61]	1%/1 mm	1.7231	1.5993	1.2690	3.0660	2.6364	2.2652	1.7408	4.4818	0.6493
		2%/2 mm	1.0709	0.7864	1.3264	5.6330	1.8495	1.2493	1.7169	6.6010	0.6314
		2%/3 mm	0.7462	0.5641	2.0615	9.2111	1.3494	0.8660	2.5374	9.3495	0.6356
Balanced Multimodality	ResNet18 [63]	1%/1 mm	1.7047	1.3624	1.2807	2.9709	2.5799	2.0178	1.6799	4.3262	0.6629
		2%/2 mm	1.0437	0.7619	1.2372	4.9718	1.7894	1.1533	1.6703	6.0951	0.6512
		2%/3 mm	0.7323	0.5817	1.7598	8.4276	1.2979	0.8949	2.2805	8.7005	0.6453
	DenseNet121 [53]	1%/1 mm	1.6554	1.4855	1.1600	3.1554	2.6145	2.1450	1.5494	4.6966	0.6631
		2%/2 mm	1.0385	0.7599	1.2611	5.3775	1.8531	1.1958	1.7133	6.7708	0.6357
		2%/3 mm	0.7446	0.5613	1.9863	9.3201	1.3767	0.8744	2.5759	9.6852	0.6224
	MobileVit [62]	1%/1 mm	1.7322	1.2182	1.2759	2.8021	2.5736	1.9130	1.6695	4.1985	0.6609
		2%/2 mm	1.0779	0.7003	1.3006	5.0196	1.7886	1.0464	1.7484	6.0630	0.6551
		2%/3 mm	0.7672	0.6119	1.7489	8.0622	1.3283	0.9018	2.2418	8.5363	0.6354
	RegNet [61]	1%/1 mm	1.6925	1.3151	1.0587	3.3435	2.6448	1.9737	1.3771	4.7989	0.6689
		2%/2 mm	1.0814	0.7307	1.4890	5.6002	1.8345	1.0826	1.8753	6.6543	0.6770
		2%/3 mm	0.7582	0.5553	2.0203	9.1534	1.3198	0.8208	2.5249	9.2636	0.6814

**Table 7:** DDP performance comparison.

Category	Method	DDP MAE (%)				DDP SSIM				$R^{2}$
		All	95–100	90–95	<90	All	95–100	90–95	<90	
Image Modality	UNET++ [53]	4.7676	5.3073	4.8217	3.5283	0.9527	0.9526	0.9518	0.9551	0.9753
	Ranking Loss [8]	4.5994	5.1528	4.6368	3.3719	0.9559	0.9555	0.9552	0.9585	0.9784
	TransQA [62]	4.6481	5.2112	4.6973	3.3718	0.9599	0.9591	0.9592	0.9634	0.9793
	RegNet [61]	5.0810	5.6554	5.1256	3.7927	0.9370	0.9347	0.9364	0.9432	0.9793
Unbalanced Multimodality	ResNet18 [63]	4.8604	5.2371	4.7999	4.2309	0.9490	0.9553	0.9531	0.9260	0.9748
	DenseNet121 [53]	4.8091	5.2819	4.7740	3.9207	0.9551	0.9609	0.9583	0.9356	0.9764
	MobileVit [62]	4.8784	5.4469	4.9394	3.5631	0.9568	0.9554	0.9561	0.9613	0.9774
	RegNet [61]	4.7814	5.3558	4.8183	3.5116	0.9554	0.9533	0.9551	0.9604	0.9805
Balanced Multimodality	ResNet18 [63]	4.3027	4.7272	4.3588	3.2953	0.9535	0.9600	0.9575	0.9367	0.9773
	DenseNet121 [53]	4.0220	4.4545	3.9942	3.1993	0.9640	0.9650	0.9656	0.9581	0.9751
	MobileVit [62]	4.4387	4.7451	4.3519	3.8250	0.9543	0.9598	0.9565	0.9453	0.9701
	RegNet [61]	4.2745	4.7580	4.3112	3.1921	0.9579	0.9581	0.9573	0.9589	0.9778

**Table 8:** Comparative analysis of computational resource utilization by deep learning baselines and the proposed **BMMQA**.

Method Name	Flops (GMac)	Param Size (M)
Unet++	63.7	15.9
TranQA	240.34	215.38
DenseNet	6.4	14.62
**BMMQA**	**6.5**	**14.72**

## Data Availability

The data supporting the findings of this study are available upon reasonable request from the first or corresponding author.

## References

[1] Meijers A., Marmitt G.G., Siang K.N.W., van der Schaaf A., Knopf A.C., Langendijk J.A., Both S. (2020). Feasibility of patient specific quality assurance for proton therapy based on independent dose calculation and predicted outcomes. Radiother. Oncol..

[2] Zeng X., Zhu Q., Ahmed A., Hanif M., Hou M., Jie Q., Xi R., Shah S.A. (2024). Multi-granularity prior networks for uncertainty-informed patient-specific quality assurance. Comput. Biol. Med..

[3] Park J.M., Kim J.I., Park S.Y., Oh D.H., Kim S.T. (2018). Reliability of the gamma index analysis as a verification method of volumetric modulated arc therapy plans. Radiat. Oncol..

[4] Huang Y., Pi Y., Ma K., Miao X., Fu S., Chen H., Wang H., Gu H., Shao Y., Duan Y. (2023). Image-based features in machine learning to identify delivery errors and predict error magnitude for patient-specific IMRT quality assurance. Strahlenther. Onkol..

[5] Kui X., Liu F., Yang M., Wang H., Liu C., Huang D., Li Q., Chen L., Zou B. (2024). A review of dose prediction methods for tumor radiation therapy. Meta-Radiol..

[6] Tan H.S., Wang K., Mcbeth R. (2024). Deep Evidential Learning for Dose Prediction. arXiv.

[7] Bi Q., Lian X., Shen J., Zhang F., Xu T. (2024). Exploration of radiotherapy strategy for brain metastasis patients with driver gene positivity in lung cancer. J. Cancer.

[8] Liu W., Zhang L., Xie L., Hu T., Li G., Bai S., Yi Z. (2023). Multilayer perceptron neural network with regression and ranking loss for patient-specific quality assurance. Knowl.-Based Syst..

[9] Li H., Peng X., Zeng J., Xiao J., Nie D., Zu C., Wu X., Zhou J., Wang Y. (2022). Explainable attention guided adversarial deep network for 3D radiotherapy dose distribution prediction. Knowl.-Based Syst..

[10] Hu T., Xie L., Zhang L., Li G., Yi Z. (2022). Deep multimodal neural network based on data-feature fusion for patient-specific quality assurance. Int. J. Neural Syst..

[11] Han C., Zhang J., Yu B., Zheng H., Wu Y., Lin Z., Ning B., Yi J., Xie C., Jin X. (2023). Integrating plan complexity and dosiomics features with deep learning in patient-specific quality assurance for volumetric modulated arc therapy. Radiat. Oncol..

[12] Huang Y., Pi Y., Ma K., Miao X., Fu S., Zhu Z., Cheng Y., Zhang Z., Chen H., Wang H. (2022). Deep learning for patient-specific quality assurance: Predicting gamma passing rates for IMRT based on delivery fluence informed by log files. Technol. Cancer Res. Treat..

[13] Li C., Su Z., Li B., Sun W., Wu D., Zhang Y., Li X., Xie Z., Huang J., Wei Q. (2025). Plan complexity and dosiomics signatures for gamma passing rate classification in volumetric modulated arc therapy: External validation across different LINACs. Phys. Med..

[14] Sun W., Mo Z., Li Y., Xiao J., Jia L., Huang S., Liao C., Du J., He S., Chen L. (2024). Machine learning-based ensemble prediction model for the gamma passing rate of VMAT-SBRT plan. Phys. Med..

[15] Huang Y., Cai R., Pi Y., Ma K., Kong Q., Zhuo W., Kong Y. (2024). A feasibility study to predict 3D dose delivery accuracy for IMRT using DenseNet with log files. J. X-Ray Sci. Technol..

[16] Xu S., Cui M., Huang C., Wang H., Hu D. (2025). BalanceBenchmark: A Survey for Multimodal Imbalance Learning. arXiv.

[17] Du C., Teng J., Li T., Liu Y., Yuan T., Wang Y., Yuan Y., Zhao H., Krause A., Brunskill E., Cho K., Engelhardt B., Sabato S., Scarlett J. On Uni-Modal Feature Learning in Supervised Multi-Modal Learning. Proceedings of the 40th International Conference on Machine Learning.

[18] Hua C., Xu Q., Bao S., Yang Z., Huang Q. (2024). ReconBoost: Boosting Can Achieve Modality Reconcilement. arXiv.

[19] Peng X., Wei Y., Deng A., Wang D., Hu D. Balanced Multimodal Learning via On-the-Fly Gradient Modulation. Proceedings of the IEEE/CVF Conference on Computer Vision and Pattern Recognition (CVPR).

[20] Xu R., Feng R., Zhang S.X., Hu D. MMCosine: Multi-Modal Cosine Loss Towards Balanced Audio-Visual Fine-Grained Learning. Proceedings of the ICASSP 2023—2023 IEEE International Conference on Acoustics, Speech and Signal Processing (ICASSP).

[21] Li H., Li X., Hu P., Lei Y., Li C., Zhou Y. Boosting Multi-modal Model Performance with Adaptive Gradient Modulation. Proceedings of the IEEE/CVF International Conference on Computer Vision (ICCV).

[22] Ma H., Zhang Q., Zhang C., Wu B., Fu H., Zhou J.T., Hu Q., Krause A., Brunskill E., Cho K., Engelhardt B., Sabato S., Scarlett J. (2023). Calibrating Multimodal Learning. Proceedings of the 40th International Conference on Machine Learning.

[23] Wu N., Jastrzebski S., Cho K., Geras K.J., Chaudhuri K., Jegelka S., Song L., Szepesvari C., Niu G., Sabato S. (2022). Characterizing and Overcoming the Greedy Nature of Learning in Multi-modal Deep Neural Networks. Proceedings of the 39th International Conference on Machine Learning.

[24] Wei Y., Hu D. (2024). MMPareto: Boosting Multimodal Learning with Innocent Unimodal Assistance. arXiv.

[25] Fan Y., Xu W., Wang H., Wang J., Guo S. PMR: Prototypical Modal Rebalance for Multimodal Learning. Proceedings of the IEEE/CVF Conference on Computer Vision and Pattern Recognition (CVPR).

[26] Wang W., Tran D., Feiszli M. What makes training multi-modal classification networks hard?. Proceedings of the IEEE/CVF Conference on Computer Vision and Pattern Recognition.

[27] Winterbottom T., Xiao S., McLean A., Moubayed N.A. (2020). On modality bias in the tvqa dataset. arXiv.

[28] Sun Y., Mai S., Hu H. (2021). Learning to Balance the Learning Rates Between Various Modalities via Adaptive Tracking Factor. IEEE Signal Process. Lett..

[29] Huang Y., Lin J., Zhou C., Yang H., Huang L. (2022). Modality Competition: What Makes Joint Training of Multi-modal Network Fail in Deep Learning? (Provably). arXiv.

[30] Du C., Li T., Liu Y., Wen Z., Hua T., Wang Y., Zhao H. (2021). Improving Multi-Modal Learning with Uni-Modal Teachers. arXiv.

[31] Wei Y., Feng R., Wang Z., Hu D. Enhancing multimodal Cooperation via Sample-level Modality Valuation. Proceedings of the CVPR.

[32] Wei Y., Li S., Feng R., Hu D. Diagnosing and Re-learning for Balanced Multimodal Learning. Proceedings of the ECCV.

[33] Yang Z., Wei Y., Liang C., Hu D. (2024). Quantifying and Enhancing Multi-modal Robustness with Modality Preference. arXiv.

[34] Miften M., Olch A., Mihailidis D., Moran J., Pawlicki T., Molineu A., Li H., Wijesooriya K., Shi J., Xia P. (2018). TG 218: Tolerance limits and methodologies for IMRT measurement-based verification QA: Recommendations of AAPM Task Group No. 218. Med. Phys..

[35] McNiven A.L., Sharpe M.B., Purdie T.G. (2010). A new metric for assessing IMRT modulation complexity and plan deliverability. Med. Phys..

[36] Du W., Cho S.H., Zhang X., Hoffman K.E., Kudchadker R.J. (2014). Quantification of beam complexity in intensity-modulated radiation therapy treatment plans. Med. Phys..

[37] Götstedt J., Karlsson Hauer A., Bäck A. (2015). Development and evaluation of aperture-based complexity metrics using film and EPID measurements of static MLC openings. Med. Phys..

[38] Crowe S., Kairn T., Kenny J., Knight R., Hill B., Langton C.M., Trapp J. (2014). Treatment plan complexity metrics for predicting IMRT pre-treatment quality assurance results. Australas. Phys. Eng. Sci. Med..

[39] Crowe S., Kairn T., Middlebrook N., Sutherland B., Hill B., Kenny J., Langton C.M., Trapp J. (2015). Examination of the properties of IMRT and VMAT beams and evaluation against pre-treatment quality assurance results. Phys. Med. Biol..

[40] Nauta M., Villarreal-Barajas J.E., Tambasco M. (2011). Fractal analysis for assessing the level of modulation of IMRT fields. Med. Phys..

[41] Zhu H., Zhu Q., Wang Z., Yang B., Zhang W., Qiu J. (2023). Patient-specific quality assurance prediction models based on machine learning for novel dual-layered MLC linac. Med. Phys..

[42] Shapley L. (1953). The value of n-person games. Ann. Math. Stud..

[43] Molnar C. (2020). Interpretable Machine Learning.

[44] Weber R.J. (1988). Probabilistic values for games. The Shapley Value. Essays in Honor of Lloyd S. Shapley.

[45] Freund Y., Schapire R.E. Game theory, on-line prediction and boosting. Proceedings of the Ninth Annual Conference on Computational Learning Theory.

[46] Wang K., Gou C., Duan Y., Lin Y., Zheng X., Wang F.Y. (2017). Generative adversarial networks: Introduction and outlook. IEEE/CAA J. Autom. Sin..

[47] Gemp I., McWilliams B., Vernade C., Graepel T. EigenGame: PCA as a Nash Equilibrium. Proceedings of the International Conference on Learning Representations.

[48] Hu P., Li X., Zhou Y. (2022). Shape: An unified approach to evaluate the contribution and cooperation of individual modalities. arXiv.

[49] Ono T., Hirashima H., Iramina H., Mukumoto N., Miyabe Y., Nakamura M., Mizowaki T. (2019). Prediction of dosimetric accuracy for VMAT plans using plan complexity parameters via machine learning. Med. Phys..

[50] Lay L.M., Chuang K.C., Wu Y., Giles W., Adamson J. (2022). Virtual patient-specific QA with DVH-based metrics. J. Appl. Clin. Med. Phys..

[51] Thongsawad S., Srisatit S., Fuangrod T. (2022). Predicting gamma evaluation results of patient-specific head and neck volumetric-modulated arc therapy quality assurance based on multileaf collimator patterns and fluence map features: A feasibility study. J. Appl. Clin. Med. Phys..

[52] Valdes G., Scheuermann R., Hung C., Olszanski A., Bellerive M., Solberg T. (2016). A mathematical framework for virtual IMRT QA using machine learning. Med. Phys..

[53] Huang Y., Pi Y., Ma K., Miao X., Fu S., Chen H., Wang H., Gu H., Shan Y., Duan Y. (2021). Virtual patient-specific quality assurance of IMRT using UNet++: Classification, gamma passing rates prediction, and dose difference prediction. Front. Oncol..

[54] Yoganathan S., Ahmed S., Paloor S., Torfeh T., Aouadi S., Al-Hammadi N., Hammoud R. (2023). Virtual pretreatment patient-specific quality assurance of volumetric modulated arc therapy using deep learning. Med. Phys..

[55] Radosavovic I., Kosaraju R.P., Girshick R., He K., Dollár P. Designing network design spaces. Proceedings of the IEEE/CVF Conference on Computer Vision and Pattern Recognition.

[56] He K., Zhang X., Ren S., Sun J. Deep residual learning for image recognition. Proceedings of the IEEE Conference on Computer Vision and Pattern Recognition.

[57] Huang G., Liu Z., Van Der Maaten L., Weinberger K.Q. Densely connected convolutional networks. Proceedings of the IEEE Conference on Computer Vision and Pattern Recognition.

[58] Mehta S., Rastegari M. (2021). Mobilevit: Light-weight, general-purpose, and mobile-friendly vision transformer. arXiv.

[59] Suwanraksa C., Bridhikitti J., Liamsuwan T., Chaichulee S. (2023). CBCT-to-CT translation using registration-based generative adversarial networks in patients with head and neck cancer. Cancers.

[60] Liu F., Liu J., Fang Z., Hong R., Lu H. Densely Connected Attention Flow for Visual Question Answering. Proceedings of the IJCAI.

[61] Xu J., Pan Y., Pan X., Hoi S., Yi Z., Xu Z. (2022). RegNet: Self-regulated network for image classification. IEEE Trans. Neural Netw. Learn. Syst..

[62] Zeng L., Zhang M., Zhang Y., Zou Z., Guan Y., Huang B., Yu X., Ding S., Liu Q., Gong C. (2023). TransQA: Deep hybrid transformer network for measurement-guided volumetric dose prediction of pre-treatment patient-specific quality assurance. Phys. Med. Biol..

[63] Yang X., Li S., Shao Q., Cao Y., Yang Z., Zhao Y.Q. (2022). Uncertainty-guided man–machine integrated patient-specific quality assurance. Radiother. Oncol..

